# Foaming of PLA Composites by Supercritical Fluid-Assisted Processes: A Review

**DOI:** 10.3390/molecules25153408

**Published:** 2020-07-28

**Authors:** Jennifer Andrea Villamil Jiménez, Nicolas Le Moigne, Jean-Charles Bénézet, Martial Sauceau, Romain Sescousse, Jacques Fages

**Affiliations:** 1 Polymers Composites and Hybrids (PCH), IMT Mines Ales, 30100 Ales, France; jennifer-andrea.villamil-jimenez@mines-ales.fr (J.A.V.J.); Jean-Charles.Benezet@mines-ales.fr (J.-C.B.); 2 Centre RAPSODEE, IMT Mines Albi, CNRS, Université de Toulouse, 81013 Albi, France; martial.sauceau@mines-albi.fr (M.S.); romain.sescousse@mines-albi.fr (R.S.)

**Keywords:** polylactic acid, biocomposite, nanocomposite, supercritical fluid, foaming

## Abstract

Polylactic acid (PLA) is a well-known and commercially available biopolymer that can be produced from different sources. Its different characteristics generated a great deal of interest in various industrial fields. Besides, its use as a polymer matrix for foam production has increased in recent years. With the rise of technologies that seek to reduce the negative environmental impact of processes, chemical foaming agents are being substituted by physical agents, primarily supercritical fluids (SCFs). Currently, the mass production of low-density PLA foams with a uniform cell morphology using SCFs as blowing agents is a challenge. This is mainly due to the low melt strength of PLA and its slow crystallization kinetics. Among the different options to improve the PLA characteristics, compounding it with different types of fillers has great potential. This strategy does not only have foaming advantages, but can also improve the performances of the final composites, regardless of the implemented foaming process, i.e., batch, injection molding, and extrusion. In addition, the operating conditions and the characteristics of the fillers, such as their size, shape factor, and surface chemistry, play an important role in the final foam morphology. This article proposes a critical review on the different SCF-assisted processes and effects of operating conditions and fillers on foaming of PLA composites.

## 1. Introduction

In many industrial fields, the development of porous and light polymer structures is of great interest. Foam structures have a wide list of properties that make them a better option for certain applications than their solid counterparts. Foams with high damping properties and thermal and/or sound insulation are used for isothermal packaging and underlays, those with high water tightness and lightness are used for porous structures floats [1]. Tridimensional foams are also developed as scaffolds in implant living tissues [2,3,4,5,6]. Foams with an evenly dispersed active pharmaceutical ingredient can act as a medium to deliver drugs progressively to the targeted organ or tissue [7]. Besides, foams can also be used to lighten structures in armament, construction, sports and leisure, and automotive industries among others.

Foams can be obtained by two main routes depending on the nature of the blowing agent, i.e., chemical or physical. Chemical blowing agents (CBAs) are organic and/or inorganic compounds that decompose thermally into gases not reacting with the polymer matrix [8]. There are a few key compounds used as chemical blowing agents around the world. These include azodicarbonamide (ADC), 4,4-oxybis benzene sulfonyl hydrazide (OBSH), p-toluene sulfonyl hydrazide (TSH), 5-phenyltetrazole (5-PT), p-toluene sulfonyl semicarbazide (PTSS), dinitrosopentamethylene tetramine (DNPT), sodium bicarbonate (SBC), and zinc carbonate (ZnCO_3_). Azodicarbonamide (ADC) is widely recognized as the leading chemical blowing agent and accounts for approximately 85% of the chemical blowing agents volume consumed within Western Europe [9]. Nevertheless, CBAs present some drawbacks: (i) the presence of residues in the produced foams limit their applications, especially in the medical industry [10]; (ii) their mostly exothermic reactions make the foaming process conditions and the final cell structure difficult to control [11]; and (iii) they are harmful for users, causing skin, eye, and airway irritation, as well as allergic reactions.

Physical blowing agents (PBAs) are compounds that expand quickly because of a phase change such as the vaporization of liquids or supercritical fluids (SCFs) at the foaming temperature and pressure [7,8]. In general, they have high foaming efficiency, they should not be toxic, and must have high heat stability. However, some of them, like hydrofluorocarbons, can be highly inflammable and may have a negative effect on the atmospheric ozone layer (e.g., Freon 11 and Freon 113). In this regard, safety precautions must be taken by the producers, and sometimes their use is limited by legislation in many countries [8]. SFCs like carbon dioxide (sc-CO_2_) and nitrogen (sc-N_2_) have emerged as the best options among the PBAs. A supercritical fluid is defined as a substance for which both pressure and temperature are above the critical value [12]. The chosen SCF must be non-toxic, non-flammable, and chemically inert, and its residues should be easily removed. By varying the pressure and temperature, their tuneable physical properties present an opportunity in modulating the polymer–SCF interactions, which represents a huge advantage over other blowing agents. In addition, SCF can be used in the most common foaming processes: batch, injection molding, and extrusion [13].

Foaming with SFCs, though conceptually simple, is a complex dynamic process requiring full appreciation of the fundamentals of thermodynamics; physics; the chemistry of solutions, interfaces, and interacting species; as well as polymer sciences and process engineering [7]; sc-CO_2_ being the most used. In general, the polymer is exposed to CO_2_ at the operating pressure and temperature, which plasticizes the polymer upon its solubilization and reduces its apparent glass transition temperature or melting point to the processing temperature [14]. Upon venting the CO_2_ by depressurization, thermodynamic instability causes supersaturation of the CO_2_ dissolved in the polymer matrix, and therefore nucleation of cells occurs. The growth of the cells, which is can be associated to a coalescence phenomenon, continues until the polymer vitrifies [14]. The saturation pressure, the saturation temperature, and the depressurization rate are the key parameters that determine the number of cells and the cell size distribution in the final foam [14]. Different works report sc-CO_2_ as a blowing agent being used with a wide range of polymers such as poly(methyl methacrylate) (PMMA) [15,16,17], polycarbonate [18,19,20,21,22], polyethylene terephthalate (PET) [23,24,25,26], polystyrene [12,27,28,29,30,31], glycol-modified PET [32,33], polyvinyl chloride (PVC) [34], polypropylene [35,36,37], polyurethane [38], polyimide [39], and polycaprolactone (PCL) [40,41,42]. Furthermore, the use of sc-CO_2_ has extended to the composites and biocomposites foaming [4,5,10,43,44,45,46,47,48], which shows the versatility of sc-CO_2_ as a foaming agent.

**Figure 1:**
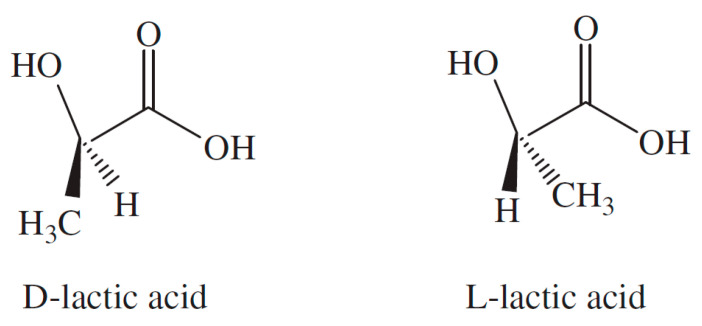
Enantiomers of lactic acid [55].

As discussed by Biron [1], most of the produced foams in the industry use petroleum-based thermoplastic matrices as raw material, but due to the shortage of fossil resources and the rise of environmental issues and related societal concerns, biopolymers (biobased and/or biodegradable and/or biocompatible polymers) are more and more used [49]. Poly(lactic acid) or polylactide (PLA) is a compostable aliphatic thermoplastic polyester typically derived from fermented plant starch as corn or sugarcane. PLA can be prepared by different polymerization process from lactic acid including polycondensation, ring opening polymerization of lactide (most common process), and by direct methods like azeotropic dehydration and enzymatic polymerization [50]. The building block of PLA, lactic acid (2-hydroxy propionic acid), can exist in optically active d- or l-enantiomers (Figure 1). Depending on the proportion of the enantiomers, PLA of variable material properties can be obtained [51]. In addition, the stereochemistry and thermal history have direct influence on PLA crystallinity, and therefore on its physical properties. PLA with PLLA content higher than 90% tends to be crystalline, while the lower optically pure is amorphous [52]. The melting temperature (*T_m_*) and the glass transition temperature (*T_g_*) of PLA decrease with decreasing amount of PLLA [53]. Its physical characteristics, such as density, heat capacity, mechanical, and rheological behavior, are dependent on its thermal transition temperatures [54].

It is well known that up until recent decades, the main uses of PLA have been limited to medical applications [50], but its interesting properties have made it the most studied biopolymer [52,55,56], and consequently generated a great amount of interest in different industrial fields [50,53,57,58]. Its use as a polymer matrix for foam production has increased, and in the first instances of its use chemical blowing agents were employed [59,60,61,62]; however, the use of PBAs, especially SFCs, increased in the different foaming processes. Different works have reported the use of SFCs as foaming agents for PLA. Nitrogen was used for injection molding [63,64,65,66,67,68,69,70,71] and batch foaming [72]. However, Li et al. [73] showed that the solubility of CO_2_ in PLA is approximately ten times that of N_2_. Therefore, the use of CO_2_ increased for the different blowing process: batch [3,11,74,75,76,77,78,79,80,81,82,83,84,85,86,87,88,89], injection molding [2,90], and extrusion [13,49,91,92,93,94,95,96].

Currently, the mass production of low-density PLA foams with a uniform cell morphology using sc-CO_2_ or sc-N_2_ as blowing agents is a challenge. This is mainly due to PLA’s low melt strength and its slow crystallization kinetics. The melt strength corresponds to a measure of the extensional viscosity and can be described as the resistance of the polymer melt to stretching. This property, which is closely related to the intrinsic rheological behavior of PLA and processing conditions, is critical during the growth phase of the cells to limit coalescence and obtain uniform cell morphology [10]. In the previously listed works, different strategies were tested in order to improve the PLA foamability: (i) introducing a chain extender to create a branched structure [97,98,99], (ii) modifying the L/D ratio of the PLA molecules [100,101], or (iii) varying the PLA molecular weight [97,100,101,102]. These macromolecular approaches proved to be efficient in improving PLA foamability thanks to enhanced crystallization rate and melt strength [13]. Likewise, compounding PLA with different types of fillers appears to be an alternative or complementary option.

Potential fillers can vary in origin from lignocellulosic fibers from different sources [103,104] to mineral ones, especially talc [105,106], clays [90,107], and silicates [108,109], as well as in terms of size, from micro- [110] to nanoparticles [109,111,112], and shape. The presence of a filler in the polymer melt can increase the cell nucleation rate during foaming, acting as a heterogeneous nucleation agent [44,45,46], and also can increase the melt strength [105,106] and the crystallization rate of PLA [113,114]. Compounding PLA with fillers does not only have foaming advantages, but can also improve the performances of the final composites. When using lignocellulosic fibers, mechanical properties of the composite such as Young’s modulus, tensile strength, elongation at break, among others, can be tailored by changing the characteristics of the fiber [103]. Nanoclay particles can improve the flame retardancy when added to a polymeric matrix [115]. Nanocellulose-reinforced nanocomposites have enhanced electrical conductivity [116,117] and mechanical properties [111], and layered silicate-reinforced nanocomposites showed enhanced tensile and flexural modulus, increased heat distortion temperature, reduction in flammability, and increased barrier for gases and liquids, among others [109].

This literature review intends to summarize and discuss the works that used the compounding of PLA with micro/nanofillers from either organic or mineral as a strategy to improve the PLA foaming behavior, focusing on the use of supercritical blowing agents with the different existing foaming process. The first part deals with the two batch foaming processes, i.e., temperature- and pressure-induced. The second part is focused on foaming injection molding (FIM). Low-pressure and high-pressure FIM are introduced. Finally, extrusion foaming is presented. In general, the effects of operating conditions; fillers in terms of size, shape, content, and surface treatment; as well as the effect of chain extenders are reviewed and discussed.

## 2. Batch Foaming

Microcellular batch processing technology was invented by Jane Martini [118], from 1980 to 1984 at the Massachusetts Institute of Technology (MIT). The first U.S. patent was filed in 1984: a polystyrene foam was produced in an autoclave using nitrogen as the blowing agent, and after saturation of the sample, it was immersed in a stirred glycerine hot bath and then submerged in cold water; argon and carbon dioxide were also tested [119]. Batch foaming of polymers is a discontinuous process carried out normally in an autoclave. The samples are saturated in a pressure vessel a certain time, and their foaming is achieved by inducing instability into the system. Pressurized gas solubility in polymers increases with pressure but decreases with temperature. Therefore, in the batch foaming process, the instability can be induced by a sudden drop in pressure (pressure quenching) or by an increase in temperature thus causing polymer foaming. Table 1 presents a list of published works using the batch foaming technique for PLA-based (nano) biocomposites with sc-CO_2_ as the blowing agent.

### 2.1. Temperature-Induced Batch Foaming

**Figure 2:**
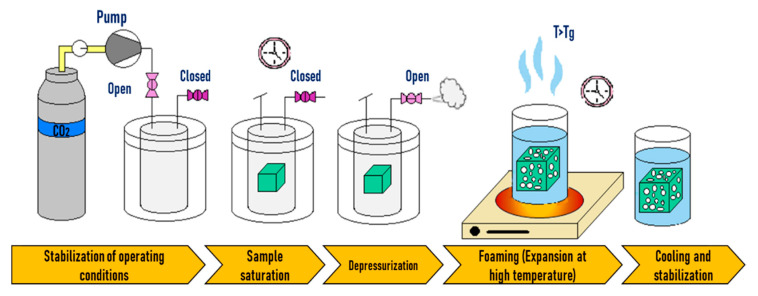
Principle of the temperature-induced batch foaming process. Adapted from Standau et al. [132].

In temperature-induced batch foaming, saturation with the blowing agent is achieved at low temperatures (10–25 °C) and low pressures (2–5 MPa). The saturated sample can be taken out of the vessel without an immediate expansion as shown in Figure 2. By plunging the saturated sample in a hot liquid such as water, glycerine, or oil, foaming is originated, the temperature (above the *T_g_*) leads to (i) an increase in the chain mobility at the same time that the polymer gets softened and (ii) a drop in the solubility of the gas in the polymer. Consequently, this results in cell nucleation and growth. To ensure the stabilization of the foam, a cooling step is necessary [132].

#### 2.1.1. Operating Conditions Effects

**Figure 3:**
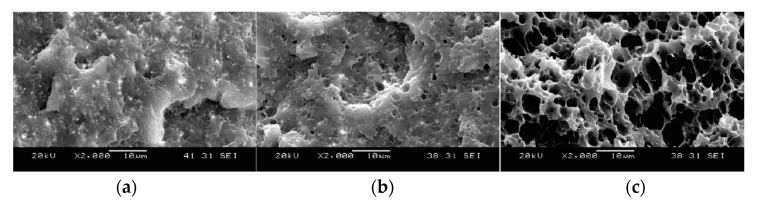
PLA/CNFs (1 wt%) foams micrographs at (**a**) 100 °C, (**b**) 110 °C, and (**c**) 120 °C. Reprinted from Ding [120].

Ding [120] studied the effect of foaming temperature on PLA/cellulose nanofibrils (CNFs) foams. The void fraction increased and foam density decreased as the foaming temperature increased. It can be seen in Figure 3 that the cell size increased with foaming temperature. At 100 °C, the diameters of most cells were below 1 µm. Both nano-sized cells and micro-sized cells were observed at foaming temperature of 110 °C. When the temperature was increased to 120 °C, only micro-sized cells were visible. Despite the fact that a relatively uniform cell size distribution was achieved in the core area of the samples, a few large bubbles were observed in the cross section of the foam. The crystallinity of the composite foams was characterized, and it was found that the degree of crystallinity decreased with foaming temperature. This was attributed to the melting of a certain fraction of crystals at high temperature in the hot oil bath during the foaming process. This fraction of melted crystals was not recovered during cooling of the PLA/CNFs foam.

#### 2.1.2. Filler Size, Shape, and Content Effects

**Figure 4:**
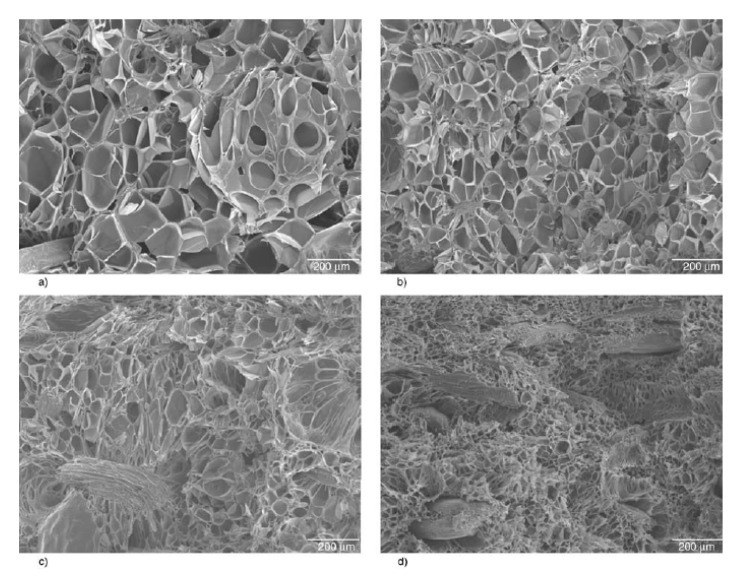
Effect of wood fiber content on the cellular structures of PLA: (**a**) 10 wt%, (**b**) 20 wt%, (**c**) 30 wt%, and (**d**) 40 wt%, saturation pressure 2.76 MPa for 4 days, magnification (100×). Reprinted from Matuana and Faruk with permission from Express Polymer Letters [123].

Rizvi et al. [122] prepared PLA/chitin nanowhisker foams. In this work, composites with chitin (PLA/C), chitin nanowhiskers (PLA/nC), and a compatibilized composite using 2 wt% of grafted maleic acid (PLA/nC-gMA) were foamed. In general, the addition of chitin resulted in a decrease in the foam density compared with the neat PLA foam. However, when varying chitin content, an ANOVA statistical test suggested that there was no significant difference between the different content’s means. Therefore, there was no significant effect of chitin content on the foam densities of PLA/C composites. For their part, Matuana and Faruk [123] studied the effect of wood flour content on the cellular structures of PLA/wood flour foams (Figure 4) All microcellular foamed PLA/wood flour composite samples had cells with finer average size than their neat PLA counterpart. The cell size decreased as the wood flour content increased in the matrix. The incorporation of wood flour into the PLA matrix produced this expected effect, as it increased the melt viscosity of the matrix and made the composites stiffer than the unfilled PLA, which provided high resistance to the cell growth in the polymer matrix. Increasing the wood flour content in the PLA matrix tended to noticeably decrease the expansion ratio of PLA in foamed samples.

In general, increasing the filler content in the PLA matrix tended to noticeably decrease the expansion ratio of PLA in foamed samples. This tendency could be attributed not only to the number of nucleated cells and their growth, which controls the volume expansion ratio (or void fraction) during the foaming process, but also to the strong dependency on the amount of gas molecules dissolved in the material as well as the volume fraction of matrix in the composite [127]. Consequently, less gas was absorbed by the composite samples during foaming compared to that absorbed by neat polymer, and the resulting expansion and void fraction were lower.

**Figure 5:**
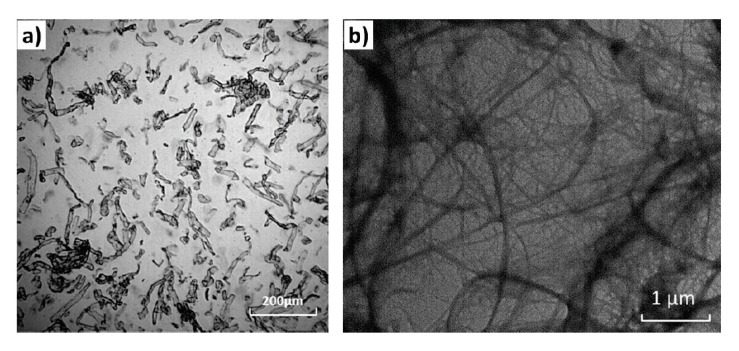
Micrographs of (**a**) micro-cellulose fibers (MFs) from optical microscopy and (**b**) cellulose nanofibrils (CNFs) from transmission electron microscopy. Reprinted from Ding [120].

Ding [120] compared the effect of cellulose nanofibrils (CNFs) and micro-cellulose fibers (MFs) (Figure 5) at different concentrations on the foam morphology. Compared with the MFs, the CNFs remarkably accelerated the crystallization of PLA, which was completed within a shorter period (~10 h vs. 20 h for MFs). At the same filler content, due to their larger surface area, CNFs also offered more nucleation sites, and thus induced a larger number of smaller crystallites compared with MFs. Due to the slower crystallization kinetics of the neat PLA and the PLA/MFs composites, the crystallization process was not completed, even when the maximum CO_2_ absorption was reached (~7 h).

**Figure 6:**
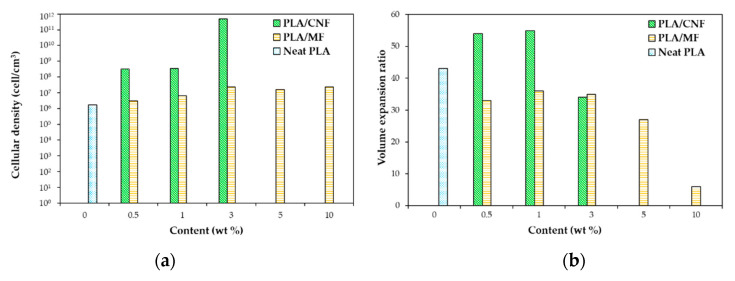
(**a**) Cellular density and (**b**) volume expansion ratio for PLA/cellulose foam composites. Data taken from Ding [120].

Figure 6 compares the cellular density and expansion ratio of the obtained foams. It can be noticed that CNFs produced foams with a higher expansion ratio and cellular density than MFs at the same content. Compared to the neat PLA, CO_2_ had a lower solubility in PLA composites, especially PLA/MF composites, which may result in lower expansion ratio (Figure 6b). The author assumed that the operating foaming temperature might have been too high for the neat PLA and PLA/MF composites. However, high cell densities and high volume expansion ratios were obtained for low CNF content composites (0.5 and 1 wt%) even with a lower amount of dissolved gas (vs. neat PLA and PLA/MF composites). This is attributed to the unique features of CNFs (i.e., large surface area and long aspect ratio), which significantly changed the thermal and rheological properties of PLA. Indeed, the increase in PLA viscosity was more pronounced with CNFs than with MFs, even at low contents such as 0.5 wt%. Besides, the higher surface area and aspect ratio of CNFs provided more interfacial area and greater interactions between CNFs and PLA chains by forming a network structure.

The cell nucleation and cell growth are two competing processes: enhanced cell nucleation by CNFs could result in a lower amount of gas available for the cell growth. Meanwhile, the increased melt strength brought by CNFs significantly prevented the cell collapse and coarsening, thus cells could be expanded to larger sizes and a higher expansion ratio was obtained. At 3 wt%, the effect of CNFs’ network structure on the restriction to the volume expansion became pronounced. Therefore, the expansion ratio decreased notably and was similar to that of the MFs.

**Figure 7:**
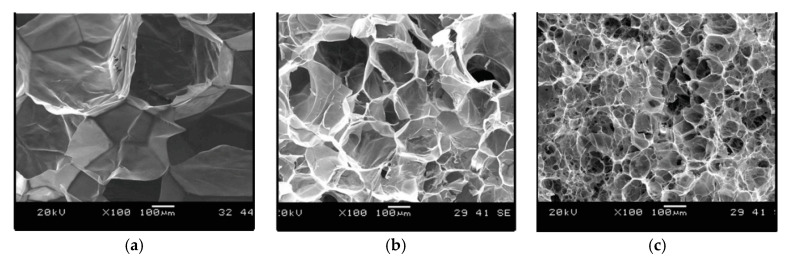
Foam morphology: (**a**) Neat PLA, (**b**) PLA/MF composites, and (**c**) PLA/CNF composites (3 wt% cellulose). Reprinted from Ding [120].

The cell structures were improved and cell size became smaller as MFs content was increased. With the use of CNFs, the foam morphology was improved further, and the cell size became smaller than that of PLA/MFs composites (Figure 7). This could be due to two main reasons: On one hand, at the same filler content, CNFs had a larger surface area, thus the number of preexisting bubbles and nucleated crystals would be greater. On the other hand, the melt viscosity of PLA/CNFs composites was considerably higher than that of PLA/MFs counterparts.

The results of this study show that compared to the PLA/MFs composite foams, the PLA/CNFs composite foams had a higher cell density (>1 order of magnitude) and a much finer cell structure. At low filler contents (0.5 and 1 wt%), PLA/CNFs composites exhibited a higher volume expansion ratio than the PLA/MFs counterparts. The differences observed between MFs and CNFs cases were attributed to the crystallization kinetics and unique features of CNFs. In particular, the cell nucleating effects of CNFs and a larger number of smaller crystals induced by CNFs resulted in higher cell nucleation rate.

#### 2.1.3. Filler Surface Treatment Effects

In the work of Qiu et al. [121], PLA/cellulose nanocrystals (CNCs) foams were the focus of the study. In this particular case, composites using 3 wt% of untreated cellulose nanocrystals (CNCs) and acetylated cellulose nanocrystals (aCNCs) with two different degrees of substitution (DS) were used (aCNCs(L) DS:0.58 – aCNCs(M) DS:1.26). The presence of both CNCs and aCNCs led to an increased expansion ratio of foamed systems, reduced cell size, and higher cell density. It is notable that the foams containing aCNCs had lower cell size and higher cell density than the ones with untreated CNCs. For the untreated CNCs filled foam, poor particle–polymer interactions led to poor dispersion of CNCs, and as a consequence the cell wall surface was penetrated with free ends of CNCs or sharp edges of large CNCs aggregates, especially around the interface, forming a rough wall surface structure. For the aCNC-filled systems, this trend weakened due to improved polymer–particle interactions. The increased expansion ratio caused by the increased nucleation efficiency yielded higher extension stress in the system, resulting in the formation of more surface defects during the process.

Regarding the mechanical properties of the foams, it was seen that PLA-CNCs foam has lower Young’s modulus and tensile strength than the neat PLA foam. The higher cell density means that there was a higher volume ratio of void in the system, and therefore a lower modulus. PLA-aCNCs(L) foam showed the same downward trend as that of PLA-CNCs. This indicates that the improved interfacial adhesion was not enough to guarantee good load transfer from the matrix to the particles in the case of a low degree of substitution. However, PLA-aCNC(M) shows approximately the same modulus as the neat PLA. As for the treated nanocellulose, having particles with a lower degree of substitution, those with moderate DS present stronger interactions with the matrix polymer, this can be confirmed by the increase in the value of the Flory–Huggins interaction parameter.

### 2.2. Pressure Induced Batch Foaming

**Figure 8:**
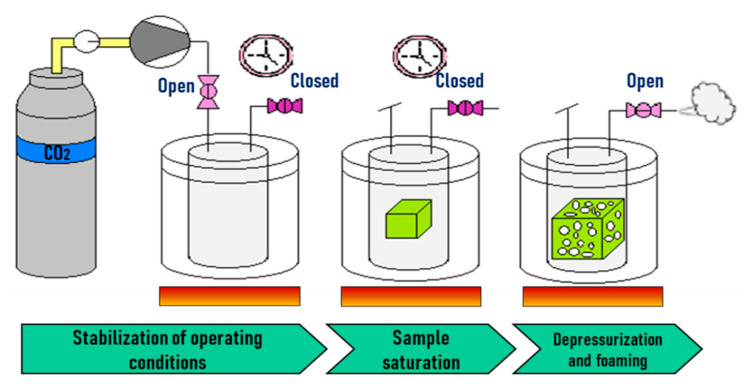
Pressure-induced foaming process. Adapted from Standau et al. [132].

In the case of pressure-induced foaming, the applied thermodynamic instability corresponds to a pressure drop, as illustrated in Figure 8 When opening a relief valve in the vessel, the pressure drops suddenly, and the heated polymer gets rapidly supersaturated and the solved gas cannot be withheld by the polymer. Then, phase separation is induced, and cell nucleation and growth take place, provoking the expansion of the sample and generating a porous structure [132].

#### 2.2.1. Operating Conditions Effects

Boissard et al. [124] foamed PLA/microfibrillated cellulose (MFC) composites at different temperatures and depressurization rates (dP/dt). Decreasing T and dP/dt resulted in reduced cell size and a narrower cell size distribution for all the materials. Coalescence was more obvious at high T and high dP/dt, resulting in significantly increased cell sizes locally. At the operating conditions, the low viscosity of the polymer melt was presumed to promote the decrease in the cell wall and, as a consequence, the gas diffusion was facilitated. When rapid stabilization was not applied, coalescence and open cell structures were favored. The most homogeneous structures were obtained with T = 155 °C and dP/dt = 4 bar/s. A similar result was obtained by Kang et al. [129], who used silk fibroin powder from silkworm cocoons to produce PLA/silk composite foams. The cell size continuously increased with the increase in the saturation temperature. Accordingly, cell density decreased.

**Figure 9:**
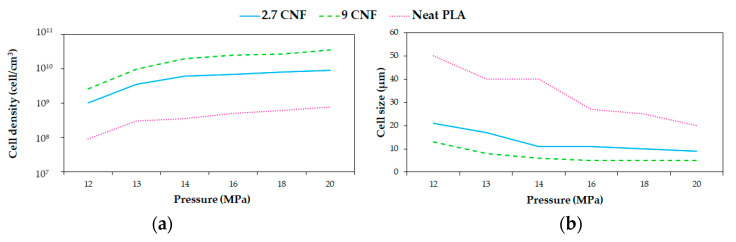
Effect of pressure in (**a**) cell density and (**b**) cell size of PLA/CNFs foams. Data taken from Dlouhá et al. [126].

Dlouhá et al. [126] foamed PLA/cellulose nanofibrils (CNFs) at pressures between 12 and 20 MPa and two different cellulose contents (2.7 and 9 wt%). The foaming temperature was chosen as 60 °C. Their results suggest that at low foaming pressures, the bulk foam density of all foams presented no difference, while at high foaming pressures, the bulk density of the nanocomposite foams with higher amounts (9 wt%) of nanocellulose was meaningfully higher compared with the other foams. This could be attributed to the differences in the rheological properties of all of the samples. Figure 9 shows that when increasing the pressure, cell density increased and the cell size decreased for both neat PLA and composites. Besides, this illustrates that at pressures above 14 MPa, the variations in pressure became less important for changing the cell morphology. The minor changes in the morphology of the nanocomposite foams when increasing the foaming pressure above 14 MPa indicated that the optimal conditions for the processing of nanocomposite foams were different from the optimal processing conditions for neat PLA foam.

**Figure 10:**
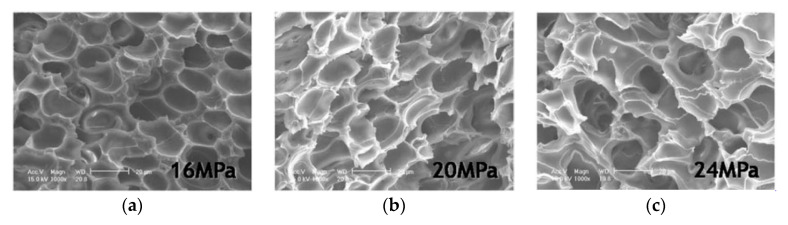
Effect of pressure on cell morphology of PLA/silk foams (7 wt%, 155 °C) at (**a**) 16 MPa, (**b**) 20 MPa, and (**c**) 24 MPa. Reprinted from Kang et al. [129]. Copyright © 2020 WILEY-VCH Verlag GmbH & Co. KGaA, Weinheim.

A study made by Ema et al. [131] using an organically modified layered silicate (OMLS) having different types of intercalants, synthesized by replacing Na^+^ ions in montmorillonite (MMT) with alkylammonium cations (octadecylammonium (ODA), showed that when increasing pressure, cell size decreased and cell density increased, for PLA and its nanocomposites; Dlouhá et al. had similar findings [126]. Figure 10 depicts the effect of pressure in the PLA/silk (7 wt%) composites produced by Kang et al. [129]. The foaming temperature was 155 °C. In this case, the cell sizes continuously increased with increasing pressure, contrary to the tendency found by the precedent authors. It can be remarked in the SEM images of 20 MPa and 24 MPa that entire cells are barely visible; in this case, it would be more appropriate to talk about the large amount of cells that collapsed and not about an increase in cell size. It is important to remark that at high temperature the coalescence became more important, which is also the case in this study.

Boissard et al. [124] prepared PLA/MFCs biocomposites containing 1 and 5 wt% MFCs (P1-P5) by using a two-step solvent-free process. A wet mixing procedure was first used to combine the PLA powder and MFCs. This was followed by a compression molding step and, for some samples, an extra extrusion step.

The samples P1 and P5, which were compounded by wet mixing and compression molding, experienced far more limited and local expansion during foaming as compared to the neat materials. This was attributed primarily to the stability of the MFCs network with strong interfibrillar interactions, which was expected to remain rigid at the operating temperature and thus hinder the expansion of the polymer. The foam expansion was six fold greater in foams obtained from the composites after extrusion, which highlights the importance of the filler dispersion within the polymer matrix to promote foaming. It was expected to obtain a reduction in the overall mechanical stiffness and effectiveness of the diffusion barrier paths of the PLA-MFC interfaces, due to the loss of continuity of the MFCs network; this is in agreement with any of the above explanations for the low expansion in the non-extruded composites. Coalescence increased with the number of processing steps and MFC content. Thus, the neat PLA foams showed a more homogeneous structure than the foams obtained with extruded PLA, which in turn showed a more homogenous structure than the PLA/MFCs composites. A contributing factor might be the decrease in viscosity due to PLA degradation during compounding, which would also have a significant impact on its foaming capacity and the final foam properties.

#### 2.2.2. Filler Size, Shape, and Content Effects

**Figure 11:**
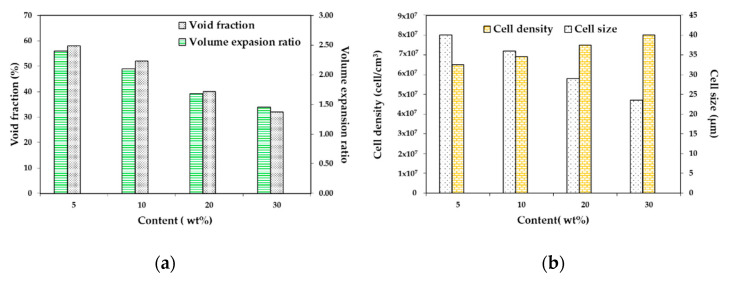
Effect of jute fiber content on the foam microstructure of the PLA/jute fiber biocomposites; (**a**) void fraction and volume expansion ratio and (**b**) cell size and cell density. Data taken from Zafar et al. [128].

Foaming of PLA and jute fiber biocomposites was carried out by Zafar et al. [128]. It can be observed in Figure 11 that when increasing the jute fiber load, the cell density increased and the cell size dropped. This may be explained by the presence of jute fibers in the PLA matrix providing heterogeneous nucleation sites. Increasing the jute fiber content drove a further increase in the melt viscosity of the matrix, which led to difficulty in cell growth and thus a reduction in average cell size. Besides, adding the jute fibers to the PLA matrix meaningfully reduced the void fraction and expansion ratio of the foamed biocomposites. A similar result was obtained by Kang et al. [129], who used silk fibroin powder to produce PLA/Silk biocomposite foams. The average cell size decreased with increasing silk load, and the cell density increased simultaneously. For a given foaming condition, the higher filler content limited gas expansion, thus leading to a foam with a lower porosity and less interconnected pores. The decreased cell size with higher filler contents could be explained by a good interface between the two components, which favored the presence of CO_2_ in the polymer matrix.

Neagu et al. [127] studied the influence of wood fiber in reinforced PLA foams. The wood fiber content in the mat was varied: 1, 5, 10, and 20%, by weight. The expansion ratio was decreased with increasing wood fiber load. A cell size gradient was observed, with the cells being larger in the center of the foams. This might have occurred because the sample is not completely in contact with the mold, so the parts in contact with it are cooled faster than the center, giving more time to the cells in the inner part to grow. Foams with 1 wt% wood fibers showed a macrostructure similar to the neat PLA foams and even higher expansion ratios. As the wood fiber load increased, the morphology changed: larger cells or pores elongating perpendicular to the foaming direction appeared. This can be explained based on the orientation of the fibers in the samples, which was uniform along the perpendicular plane of the non-foamed specimen. Foams with up to 10 wt% content of wood fibers also look like conventional foams with more or less regular cells, even if the upper parts of the foams were porous and the bottom parts were much denser. At 20 wt% wood fiber content, the material seemed to be only a stacked layer of commingled samples. In general, the authors suggest that in order to obtain foams with homogeneous morphology, the operating conditions must be adapted for different wood fiber contents. Cho et al. [125] studied the effect of cellulose nanofibrils (CNFs) and found that the average cell size of the PLA foams decreased with increasing CNFs content due to the increased viscosity, which hindered cell growth in the polymer matrix. When talking about gas solubility and diffusivity, it might be noted that these phenomena occur in the amorphous zone of the polymer, therefore, in the case of an amorphous polymer, an increase in the volume fraction of crystallites will reduce the amorphous volume, and thus the solubility and diffusivity of the gas in the matrix. DSC thermograms of PLA/CNFs nanocomposites, showed an increase in the degree of crystallinity when increasing the amount of CNFs. Consequently, the incorporation of CNFs affected the rheological properties and the crystallinity of the PLA/CNFs nanocomposites, which led to different cellular morphology of the PLA foams. Foam density of neat PLA was higher than that of the nanocomposites with 1 and 3 wt% of CNFs, owing to its weak melt strength acting to resist the cell expansion. On the other hand, the cell structures of the nanocomposite with 5 wt% CNFs foam exhibited the highest foam density.

#### 2.2.3. Filler Surface Treatment Effects

**Figure 12:**
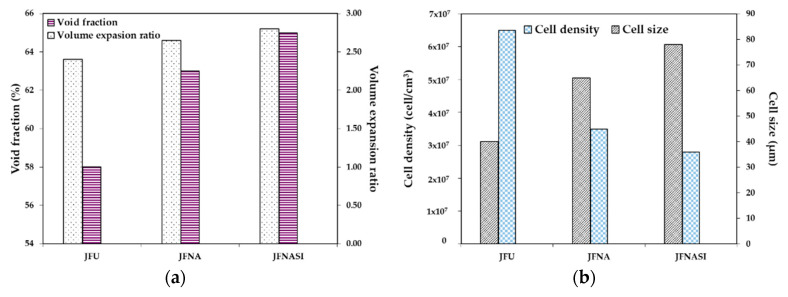
Effect of jute fiber surface treatment on the foam microstructure of the PLA/jute fiber biocomposites: (**a**) void fraction and expansion ratio (**b**) cell size and cell density. Data taken from Zafar et al. [128].

Foaming of PLA and jute fiber biocomposites was carried out by Zafar et al. [128], and fiber surface treatment on foam morphology was evaluated. PLA/jute fiber (5 wt%) biocomposites with untreated jute fibers (JFU), NaOH-treated jute fiber (JFNA), and (NaOH + silane)-treated jute fibers (JFNASI) were prepared. The amount of CO_2_ present in the PLA matrix was strongly dependent on the nature of the matrix–fiber interfacial adhesion. In PLA/JFU biocomposites, untreated jute fibers provided weak matrix–fiber interfacial adhesion, which produced a channel through which the CO_2_ can quickly escape from the matrix to the outer environment. In PLA/JFNA biocomposites, NaOH treated jute fibers allowed stronger matrix–fiber interfacial adhesion. The interfacial adhesion in PLA/JFNASI biocomposites was the strongest due to the (NaOH+silane)-treated jute fibers. As the interfacial adhesion between jute fibers and PLA increased, the concentration of CO_2_ increased in the matrix, enlarging cell size and lowering the cell density. The void fraction and expansion ratio of the foamed biocomposites increased from JFU to JFNA and then to JFNASI, as seen in Figure 12.

Neagu et al. [127] studied wood fiber-reinforced PLA foams. The wood fiber content was varied—1, 5, 10, and 20% by weight—and foams were treated with (i) butyl tetracarboxylic acid (BTCA) and (ii) with BTCA and an additional surfactant, that is, disodium hydrogen phosphate (Na_2_HPO_4_), composed of a positively charged head and a negatively charged tail. The BTCA can be used as cellulose cross-linking agent, which introduced cross-links inside the cell wall leading to increased fiber stiffness. With the surfactant, the fibers became negatively charged, which was expected to impede the aggregation of the wood fibers by reducing their ability to form hydrogen bonds. With all fiber treatments and loads from 5 to 10% there was an increase in density. This effect was slightly higher for SCWF, which was treated in a different way, allowing formation of a weaker wood fiber network in the sample. This affected the foam expansion, which was higher than that of CLWF and UWF foams.

Acetylation of the CNFs surface was performed by Dlouhá et al. [126], with the principal aim of improving the dispersion of CNFs and its interaction with PLA matrix, thus enhancing the nucleating efficiency of the CNFs particles. Optical characteristics of the nanocomposites were characterized, and it was demonstrated that the dispersion of ac-CNFs in the PLA matrix was more homogeneous compared with native CNFs. Foaming was carried out in a range of pressures between 12 MPa and 20 MPa. Treated fibers were shown to have a more important influence on the reduction of cell size and the increase in cell density than untreated ones.

**Figure 13:**
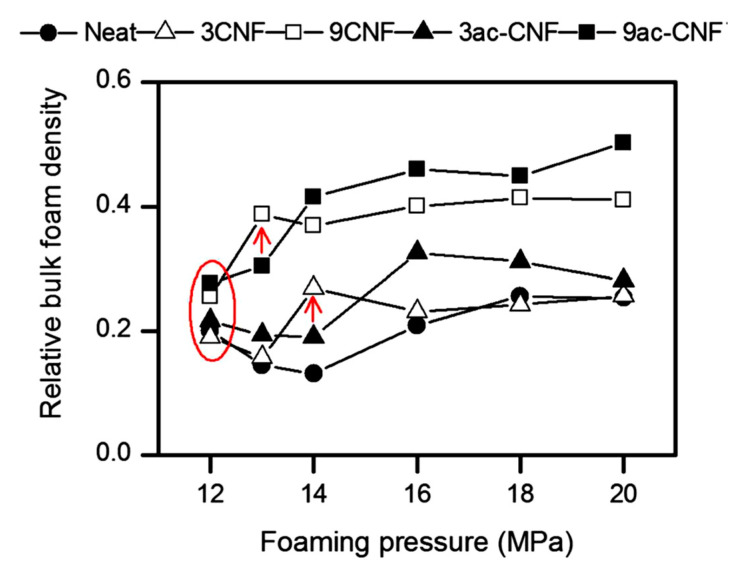
Variations of the relative bulk foam density of PLA foams as functions of foaming pressure. Arrows indicate the foaming pressure at which the relative bulk foam density increases suddenly for the CNFs composites. Reprinted from Dlouhá et al. [126]. Permission conveyed through Copyright Clearance Center, Inc.

**Figure 14:**
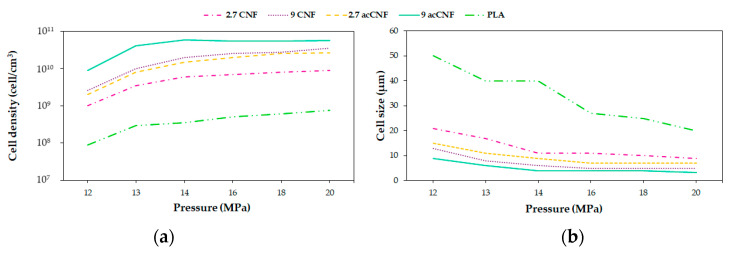
Effect of variations in pressure on the (**a**) average cell diameter and (**b**) cell density of the neat PLA and PLA/aCNFs nanocomposite foams. Data taken from Dlouhá et al. [126].

Figure 13 illustrates the influence of pressure and surface treatment on the relative bulk density of the obtained foams. At 13 MPa, an increase in density can be remarked for the 3ac-CNFs foam and only at 14 MPa in the 3CNFs foam, in the case of both treated and untreated composites with a load of 9 wt% of fibers the same trend is obtained (pointed out by arrows in Figure 13). This result is in opposition with the suggested increase in the nucleating efficiency of the CNFs surface after acetylation obtained from the melt elasticity measures. At higher foaming pressures, the rheological properties of the composite will decide the time for the foaming process, which will slow down the cell growth and impart higher bulk foam densities in the ac-CNFs foams. The better dispersed fibers in the matrix generated by the surface treatment increased the nucleation sites at a given CNF content. Looking at the relative effect of acetylated/native CNFs on the cell size and cell density (Figure 14), it can be see that the cell density in the ac-CNF composites is on average 2.7 times higher, and the cell size is 0.7 times smaller compared with the foams containing native CNFs, regardless of the CNF content.

#### 2.2.4. Use of Chain Extender Effects

Boissard et al. [124] prepared PLA/MFCs biocomposites containing 1 and 5 wt% MFCs. The chain extender Joncryl ™ ADR-4368 from BASF (Germany), an epoxystyrene-acrylic oligomer with a weight average molar mass (M_w_) of 6800 g/mol, was used. This led to much finer and more homogeneous foam structures in all the samples in spite of the high temperature used in this case. Moreover, the cell size was significantly reduced in the composite foams. The use of the chain extender counteracted the effects of degradation resulting from the multiple processing steps necessary to produce composite foams. The foam densities were found to be approximately 60–75% lower for the materials incorporating the chain extender, reflecting its positive effect on the foaming characteristics of PLA and its composites.

Wang et al. [130] studied the effect of chain extender (CE) content on cell morphology of PLA/wood flour composites foams. A constant content of wood flour of 20 wt% was chosen, and a multifunctional epoxide-based chain extender, composed of a styrene-acrylic oligomer, was used. Composites with CE (0, 0.5, 1.5, 2.5 wt%), talc (4 wt%), and lubricant (2 wt%) were prepared.

**Figure 15:**
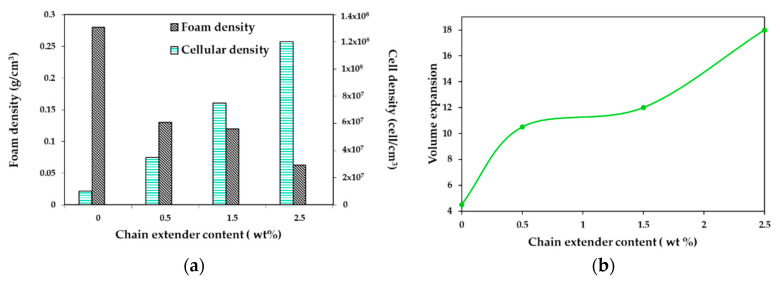
Foam characterization of PLA/wood flour composite foams: (**a**) Foam density and cell density, and (**b**) expansion ratio as a function of CE content. Data taken from Wang et al. [130].

Figure 15 depicts the density, expansion ratio, and cell density of the foamed samples as a function of the CE content. The volume expansion ratio shows an increase with increasing CE concentration (up to 18 times when 2.5 wt% of CE was added). Consequently, the apparent density decreased. A further drawback of PLA is that it has poor thermal stability and could undergo chain-scission during processing. Coalescence, cell rupture, and collapse were observed when CE was not used; this could happen because, during the foaming process, the melt could not tolerate the strain induced by the cell expansion and growth. With the use of a chain extender, the PLA formed a branched structure which improved the melt strength, thus reducing cell coalescence and avoiding cell collapse; most of the bubbles during cell growth could preserve its form, which in turn increased the expansion ratio and reduced the apparent density of composite foams.

Cell density mainly depended on the number of nucleation sites and the solubility of sc-CO_2_ in the polymer. The content of wood flour and Talc were kept constant. However, the cell density increased in an almost linear way with the CE content, showing the positive effect of CE on the efficiency of global nucleation. The authors assumed that the sites where the CE coupled with the PLA end group promoted heterogeneous nucleation, so an increase in CE concentration increased the nucleation sites, and, as a result, the cell nucleation density increased. Moreover, the CE lowered the crystallinity of PLA/wood flour composites, improving the solubility of sc-CO_2_ in the PLA, thus increasing the cell density.

**Figure 16:**
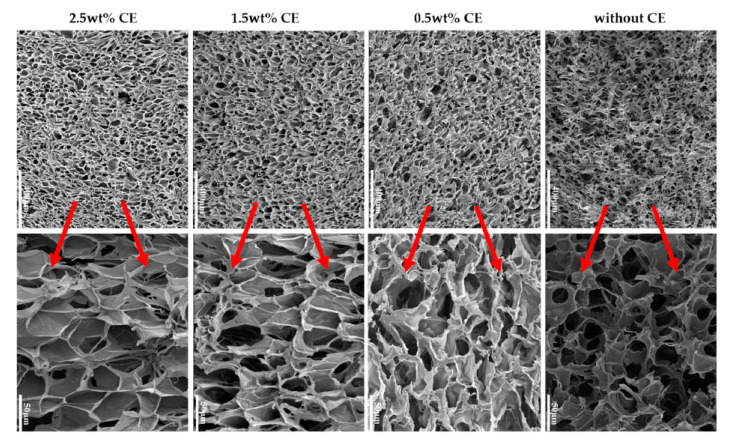
SEM micrographs of the polylactide/wood flour composite foams and the corresponding cell size distribution in the presence of various CE concentrations. Reprinted from Wang et al. [130].

CE showed a significant effect on the cell structure of the composite foams (Figure 16). A dramatic cell rupture was observed when CE was not present, which led to a high open-cell ratio caused by the poor melt strength of PLA. A cell wall thinning was obtained when adding 0.5 wt% and a better morphology was achieved. However, due to the lack of resistance of the composite to stretching, the cells still displayed an abnormal shape. Further increasing the extender content from 1.5 wt% to 2.5 wt %, the cell morphology was much better. Cells developed a polygon shape and it can be said that at this concentration, the chain extension reaction occurred to ensure that most of the PLA chains had a branched structure, leading to a higher melt strength and strain-hardening behavior during the foaming process. According to the authors, the biaxial extension stress around each cell was uniform, so the foamability was markedly improved and the final cell adopted a regular shape.

### 2.3. Conclusions

This review of state-of-the-art PLA composite synthesis under batch foaming assisted by SFCs evidences that the relationship between the final foam morphology and the nature of the thermodynamic instability is not straightforward.

**Figure 17:**
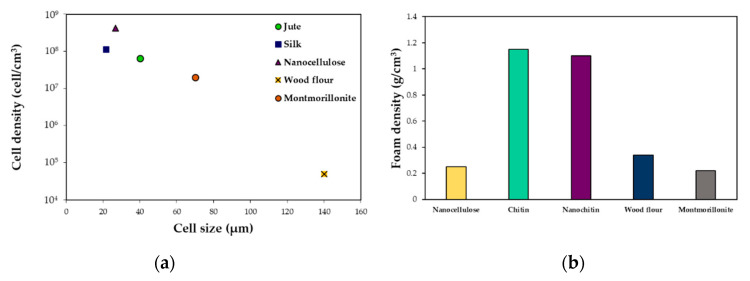
(**a**) Cell density and cell size for different fillers at 5 wt% content in PLA and (**b**) foam density for different fillers between 3 and 5 wt% contents in PLA. Data taken from Zafar t al. [128], Kang et al. [129], Cho et al. [125], Neagu et al. [127], and Ema et al. [131].

In general, when adding a filler to the polymer matrix the morphology of the foam was improved. Increasing the filler content led to an increase in cell density and a reduction in cell size and expansion ratio. Nanoparticles have shown to be a better option to obtain low-density foams compared to the samples produced with microparticles as showed by Rizvi et al. [122] and Ding et al. [120]. Applying a chemical surface treatment on the fillers to enhance polymer/filler interactions produced foams with smaller cell sizes, higher cell densities, and lower bulk densities than their non-treated counterparts. As can be seen in Figure 17 the nature of the filler and its characteristics are important parameters for the final quality of the foam in terms of cell density and size and foam density. In the different reviewed works, at contents between 3 and 5 wt%, the highest cell density of 5×10^8^ cells/cm^3^ was obtained with cellulose nanocrystals. The smallest cell size was ~21 µm in the montmorillonite loaded foams, and the less dense foams were produced when using cellulose microfibrils (0.22 g/cm^3^). Besides, adding a chain extender lead to better cell morphologies, with thinner cell walls and regular cell shapes.

Operating conditions also play a key role. When increasing the foaming temperature, foam characteristics such as cell size and void fraction increased, while foam density and cell density decreased due to coalescence. When increasing pressure, cell density increased and cell size decreased. Related to the process, the foams obtained by pressure quenching presented a higher cell density and lower cell size and foam density than the foams obtained by a thermic shock. These results provide evidence that pressure and temperature are strongly linked, and that for each filler and each process there is an optimal combination of operating conditions to obtain a foam having a uniform morphology and with the desired properties.

## 3. Foam Injection Molding

Generally speaking, foam injection molding (FIM) is quite similar to injection molding but is carried out with a blowing agent and requires some specific tools such as a special nozzle. This technique was first developed in 1997 by Trexel Inc. in collaboration with Engel Canada, when the first injection molding machine with a plunger for injection and extruding screw for plasticizing and gas dosing was developed [133]. The industry easily understood the needs of users and several alternatives with different concepts for blowing agent incorporation and mold were developed, i.e., Optifoam^®^, Ergocell^®^, Profoam^®^, and MuCell^®^. The latter has been the most popular in the industry. MuCell^®^ technology was developed by Trexel Inc., and has been employed when foaming with SCFs as the blowing agent. This technology uses a reciprocating screw as the SCF dosing element, and the SCF is injected into the reciprocating screw through the barrel [134]. It makes full use of the shearing and mixing functions of the screw to quickly complete the SCF dosing and to maintain the minimum dosing pressure in the barrel and screw for possible continuation of the process of microcellular injection molding [133].

Two options have been developed for the incorporation of the blowing agent. Either the blowing agent is dosed with the polymer pellets in the feeder, which is the case of the chemical blowing agents, or it is directly injected into the polymer melt in the barrel, which is employed for SCFs. In principle, the gas–melt mixture is conveyed by the screw towards the mold through a rotation movement. In order to accumulate the gas-loaded melt at the tip and, the injection is eased into the mold and the screw moves backwards, which is accompanied by a subsequent forward movement. Two mold concepts are in use, namely low- and high-pressure foam injection molding [132].

Volpe et al. [70] concluded that longer flow paths and faster cycle times can be obtained compared to usual injection molding due to the plasticization effect induced by the added supercritical blowing agent (i.e., the glass transition and melting temperature are decreased and the melt viscosity is reduced). Guanghong et al. [135] reviewed microcellular foams having uniform cell diameters of 1 to 100 µm, and Xu et al. [133] reviewed cell densities of 10^5^ to 10^9^ cells per cubic centimeter for foams produced by this technique.

Few works using the FIM technique for processing PLA composites using SFCs as the blowing agent have been reported. Table 2 presents the operating conditions and general information about the different published studies.

### 3.1. High-Pressure FIM

**Figure 18:**
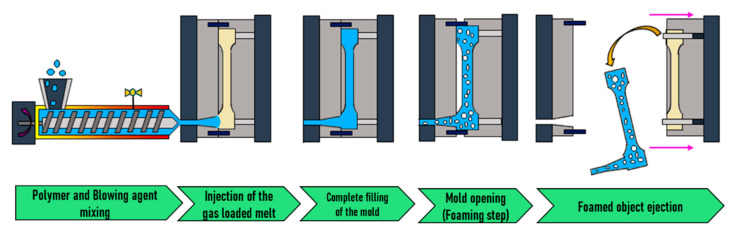
Principle of high pressure foam injection molding. Adapted from Standau et al. [132].

As illustrated in Figure 18, during this kind of FIM, the mold is entirely filled under high pressure with the gas loaded melt. Then, foaming is originated by a pressure drop generated by the partial opening of the mold, which leads to the expansion of the mold content. This method is also known as “breathing mold” or “full shot”.

#### 3.1.1. Mold Opening Speed Effects

Xie et al. [136] investigated the influence of mold opening speed on PLA/nanoclays foams. For this work, particles having a density of 1.7 g/cm^3^ were incorporated in the polymer at a content of 5 wt%. Nitrogen was employed as the blowing agent. The dropping rate of pressure caused by the mold opening is a key parameter for foaming with this technique. Four speeds of mold opening (150, 100, 90, and 80 mm/s) corresponding to four measured dropping pressure speeds (1.15, 0.9, 0.75, and 0.5 MPa/s) were studied.

**Figure 19:**
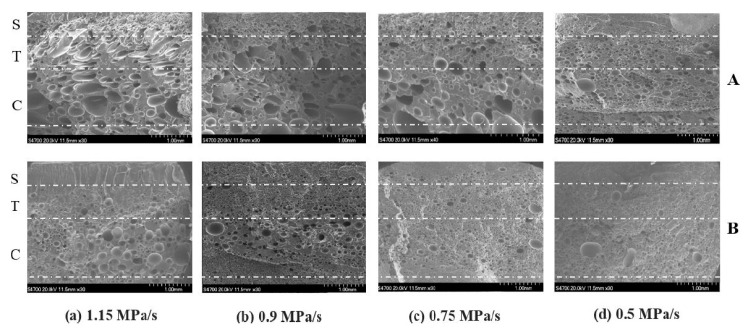
Representative SEM micrographs of the injected foams of pure PLA (**A**) and PLA/nanoclays (**B**) with four different dropping pressure speeds (1.15, 0.9, 0.75, and 0.5 MPa/s). Reprinted from Xie et al. [136].

Figure 19 shows the SEM micrographs obtained for pure PLA and PLA/nanoclays at different mold opening speeds. A cross section of all the foamed samples allowed identification of the three different regions. These regions were a skin layer, a transition region, and a core, denoted by the authors as S, T, and C, respectively. The skin layer presented a solid unfoamed structure, and the results indicated that different mold opening speeds did not change the thicknesses of the skin layer. Spherical shape cells with greater cell density and smaller cell sizes were obtained in the core region.

**Figure 20:**
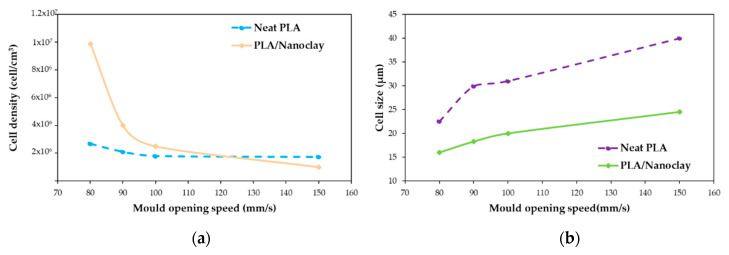
(**a**) Cell density and (**b**) cell size of foams obtained at different mold opening rates for neat PLA and PLA/nanoclays. Data taken from Xie et al. [136].

Both transition and core regions possessed a cellular structure with different morphologies. The most remarkable difference between these regions was the degree of cell elongation, which was much more pronounced in the core region, while a significant number of cells in the transition region retained their spherical shape. The shape of the cells started to be distributed with more homogeneity in terms of size and location in both regions when the speed of mold opening was decreased; this also affected the degree of cell elongation, which decreased. At lower mold opening speeds, smaller cell sizes and greater cell densities were obtained (Figure 20). It can be observed that cell size and cell density have the same trend for both PLA and PLA/nanoclays foams, but noticeable higher cell density and lower cell size were obtained for PLA/nanoclays, especially at low mold opening speed. Xie et al. [136] explain that at a higher speed of mold opening, there is a greater pressure drop and lower melt pressure, and therefore more cell nucleation and more severe collapse occurred. On the other hand, slower mold opening speed provided adequate conditions for the melt to be maintained at higher pressure and to prevent a larger number of cell nucleation sites from growing and coalescing.

Xie et al. [136] evaluated the effect of mold opening speed on foams mechanical properties. Tensile strength and modulus tended to decrease when increasing the mold opening speed, but the Izod impact strength of PLA and PLA/nanoclays foams did not change.

#### 3.1.2. Effects of Nanoclays

From Figure 19 it can be concluded that the cell structure was influenced by adding nanoclays. The cells were relatively smaller and more uniform in size and distribution, also the retention of the spherical form was higher. Up to 5%wt of nanoclay, small-sized and high-cell density foams were obtained at the same rate of mold opening speed in comparison with the neat PLA.

Regarding the foam’s mechanical properties, the addition of nanoclays improved the tensile properties of PLA foams. The tensile strength of the foamed composites samples increased up to 15% compared to foamed neat PLA as well as the modulus, which was higher particularly at the 100 mm/s (50% higher) and 90 mm/s (44% higher) speeds. These increased tensile mechanical performances were attributed to the uniformly distributed fine cells with greater density and smaller size, which in turn is due to the induced crystals caused by the presence of nanoclays, acting as an effective nucleating agent and increasing the PLA low melt strength [139]. Compared with pure PLA, the nanocomposite foams had a higher impact strength. This could be due to two main factors: First, according to Xu et al. [139], the addition of nanoclays could enhance the Izod impact strength by increasing the PLA crystallinity. Second, a larger number of smaller-sized cells uniformly distributed in the matrix should promote energy absorption during impact.

#### 3.1.3. Shape Factor and Filler Chemistry Surface Effects

Different works suggest that the surface chemistry of fillers would affect the crystal nucleating ability of the polymer matrix [140,141,142], which is important in the foaming process. Table 3 shows the chemical characteristics of cellulosic fibers used by Ding et al. [138] in their work. Northern bleached softwood Kraft (NBSK) and medium density fiberboard (MDF) fibers were compounded with PLA. NBSK fibers had smaller diameters and higher aspect ratios than MDF fibers. Ding et al. [138] studied the morphology of PLA/cellulosic-fiber composite foams manufactured using foam injection molding with N_2_ as the blowing agent. A content of 20 wt% of fibers were used. Poly (ethylene glycol) (PEG) was used as a lubricant.

**Figure 21:**
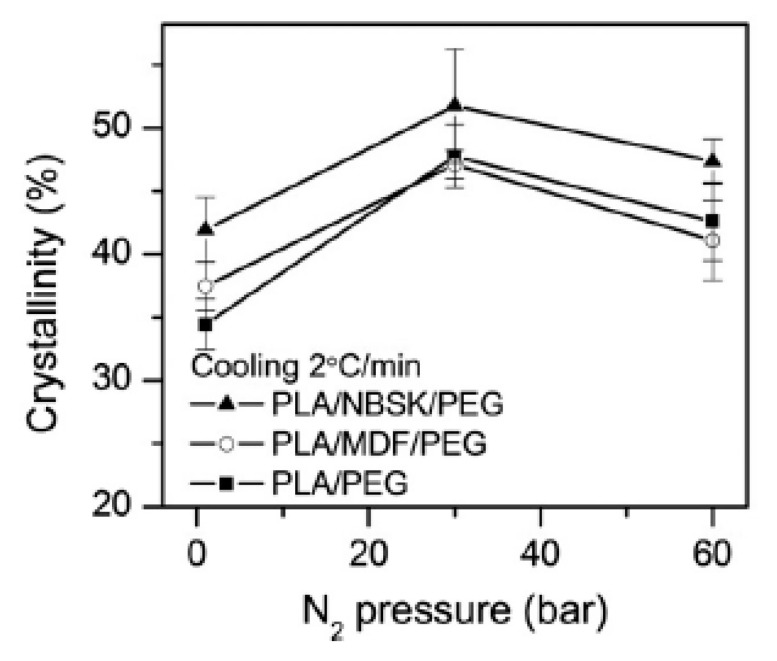
Degree of crystallinity of the PLA composites at a cooling rate of 2 °C/min under various N_2_ pressures. Reprinted from Ding et al. [138]. Copyright (2020), with permission from Elsevier.

Compared to the PLA/NBSK/PEG foam, the cell density was higher for the PLA/MDF/PEG foam. Considering their shape factor, at the same weight concentration, the higher shape factor of NBSK fibers displayed a larger surface area than that of the MDF fibers. As shown in Table 3, the hemicelluloses, lignin, and extractives contents of MDF fibers are significantly higher than those of the NBSK fibers that underwent a bleaching process during production. The presence of these non-cellulosic components (hemicelluloses, lignin, and extractives) in MDF fibers provided a different surface chemistry with less cellulose hydroxyl groups than NBSK fiber surfaces. Thus, the interactions between the weak hydrophilic PLA polymer matrix and strong hydrophilic NBSK fibers were possibly weaker than those with less hydrophilic MDF, leading to a different foam morphology. Figure 21 shows that NBSK fiber composites had a higher crystallinity compared to the MDF and PLA ones. The remarkably high viscosity measured at low frequencies for the composites charged with the NBSK fibers suggested that the fibers could have formed a 3-D network structure. Indeed, a viscosity 20 times higher was reported for the molten PLA/NBSK/PEG composites compared to that of the molten PLA/MDF/PEG composites at 180 °C. The authors suggested that the NBSK fiber network had a negative effect on cell nucleation due to the smaller pressure drop variations generated by the network when the system is subjected to a rapid pressure drop (fast mold opening).

### 3.2. Low-Pressure FIM

**Figure 22:**
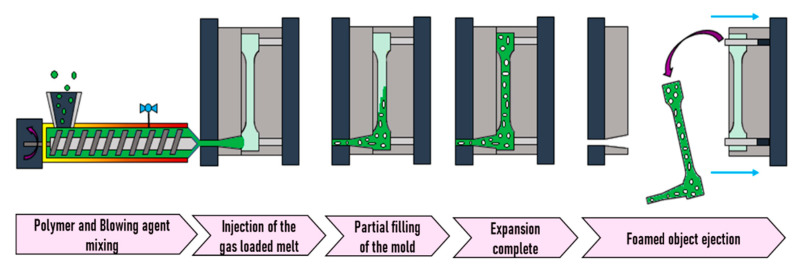
Principle of low pressure foam injection molding. Adapted from Standau et al. [132].

In low-pressure FIM (also referred to as “short shot”), the mold is only partially loaded with the mixture gas/polymer melt, which, when entering the mold, instantly suffers a pressure drop. Instantly, foaming occurs and the mold becomes fully filled expanded material as shown in Figure 22.

#### 3.2.1. Filler Size and Content Effects

**Figure 23:**
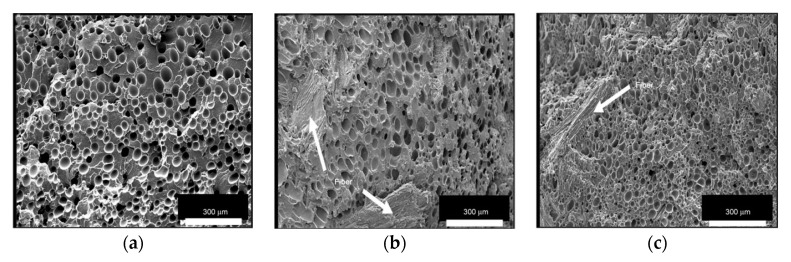
Scanning electron microscopy (SEM) image of foamed samples: (**a**) virgin PLA, (**b**) PLA/willow-fiber (80/20), and (**c**) PLA/willow-fiber (70/30). Reprinted from Zafar et al. [137]. With permission from Express Polymer Letters.

Zafar et al. [137] studied the effect of willow fibers (biomass-willow) on PLA foams. N_2_ was used as the supercritical blowing agent. Two different contents of willow fibers were tested: 20 wt% and 30 wt%. Figure 23 shows SEM images of the microcellular foam structure of neat PLA and composites. Foamed samples of composites showed cells with finer average sizes in comparison with virgin PLA foam. The cell density increased when adding the fibers and was further enhanced as the willow fiber content increased. On the other hand, increasing the fiber content increased the melt viscosity, provoking a matrix hardening compared with virgin PLA. Therefore, the cell growth became difficult, which led to reduced average cell size.

**Figure 24:**
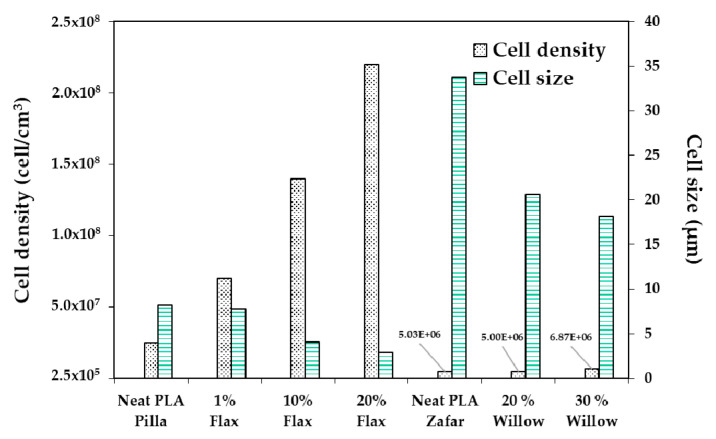
Effects of flax and willow fibers content on PLA foam cell density and cell size. Data taken from Zafar et al. [131] and Pilla et al. [105].

Zafar et al.’s [137] results are in agreement with Pilla et al.’s [105] findings. In the latter work, PLA was compounded with flax fiber to study their effects on foam cellular morphology. Composites had a flax fiber content of 1, 10, and 20 wt%. Moreover, N_2_ was used as the blowing agent. Figure 24 compares Pilla’s and Zafar’s results, and shows that with both fillers, foam morphology followed the same trend, i.e., increasing cell density and decreasing cell size with the addition of fibers. In addition, Zafar et al. [137] reported that the cell morphology of the produced foams is more uniform at higher fiber content than lower fiber content and pure PLA based on a statistical test using the standard deviation. Pilla et al. [105] reported a typical solid skin layer near the polymer–mold interface where cells were not visible due to rapid cooling of the material and the SCF escaping through the surface, which hampered cell growth. Some of the cells in the intermediate region appeared spherical on the cross section perpendicular to the flow, but they were actually more elliptical due to strong shear. Furthermore, Zafar et al. [131] reported a decrease in the void fraction and expansion ratio by increasing the willow fiber content. This trend may be expected as the volume expansion ratio and the void fraction during the foaming process are controlled not only by the number of nucleated cells, but also by the amount of gas dissolved in the matrix [123]. Indeed, increasing the filler content results in a lower volume fraction of the matrix in composites. As a result, the amount of gas absorbed in composites noticeably lowered in comparison to neat matrix [143], thus explaining the decreased volume expansion ratio and void fraction.

Zhao et al. [90] employed 4% nanoclay fibers and obtained a sandwich foam structure with small cells near to the skin layer, whereas larger pores could be found towards the center. This is in agreement with the findings of Najafi et al. [144], who used azodicarbonamide as a blowing agent for the foaming of PLA/clays by a high pressure injection molding process. Zhao et al. [90] obtained a foamed core with large gas pockets, which increases with nanoclays content and two poor foamed skin layers. A possible explanation given by the author for this kind of sandwich structure is the orientation of the clay platelets into the polymer matrix; they tended to disperse and agglomerate parallel to the skin layer due to the shear and fountain flow effects of the injection molding process. Most clay platelets existed as stacks, layers, or tactoids and served as nucleation agents. The cell density also increased significantly due to the shear stress along the mold cavity [145]. As soon as the nucleation sites were created, the cells were frozen owing to rapid cooling near the mold surface, whereas cells at the center of the cavity continued to grow and coalesce due to a much slower cooling rate.

Zafar et al. [137] studied the effect of wood fibers on the mechanical properties of PLA foamed composites. Foamed composites showed a decreased specific flexural strength and specific tensile strength when compared with their unfoamed counterparts. This decreased strength of the foamed composites might be due to the presence of cells inside the matrix. Presumably, these cells became points of stress concentration which decreased the strength of the foamed composites. A similar observation had also been reported by Kramschuster et al. [146]. The specific notched impact strength of the foamed composites showed an increasing trend when compared with their unfoamed counterparts. It was likely due to the presence of microcells, which helped in preventing the crack propagation process and absorbed the energy, thus increasing the total energy required to propagate the crack. Pilla et al. [105] reported that the specific tensile modulus of the solid and microcellular samples increased with the fiber content in the case of flax fiber-reinforced PLA composite foams. As found by Zafar et al. [137], the specific strength of both solid and microcellular composite samples was slightly lower than that of their solid and microcellular pure PLA counterparts.

#### 3.2.2. Filler Surface Treatment Effects

**Figure 25:**
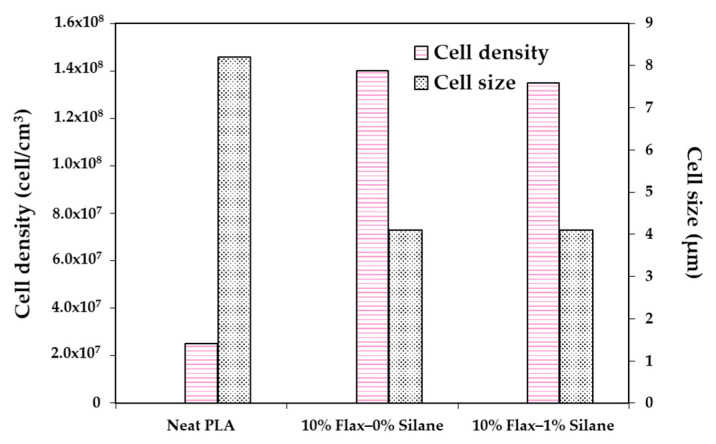
Average cell size and cell density of microcellular PLA and PLA-flax fiber composites. Data taken from Pilla et al. [105].

Pilla et al. [105] compounded PLA with 10% wt. flax fibers treated with 1% wt. silane to study the effects of the surface treatment on cell morphology. Nitrogen was used as the blowing agent. As illustrated in Figure 25, silane did not have any effect on the average cell size or cell density of the PLA/flax foams. According to the DSC analysis, the degree of crystallinity did not change when silane treatment was applied. The specific toughness and strain-at-break of the solid samples increased with silane treatment while it did not show much effect on the toughness and strain-at-break in the microcellular samples. It was also reported that silane treatment did not affect the glass transition temperature for both solid and microcellular samples.

#### 3.2.3. Pre-Foaming Effects

**Figure 26:**
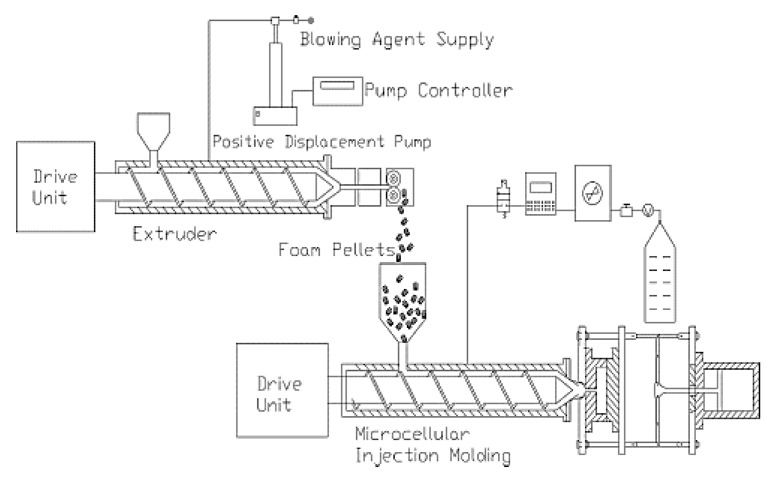
Schematic of microcellular injection molding combined with the pre-foaming extrusion process developed by Zhao et al. Reprinted from Zhao et al. [90]. Copyright (2020) American Chemical Society.

Zhao et al. [90] developed a pre-foaming step with SCF-assisted extrusion before the microcellular injection molding process (MIM) (Figure 26). They investigated the effect of the pre-foaming step on the morphology of PLA and PLA/clays foams. Organomodified montmorillonite (MMT) Cloisite 30B (C-30B) containing a methyl bis-2- hydroxyethyl ammonium quaternary salt with a cation exchange capacity (CEC) at a content of 4 wt% was used. Supercritical CO_2_ was used as the blowing agent for the extrusion process and N_2_ for the injection one.

**Figure 27:**
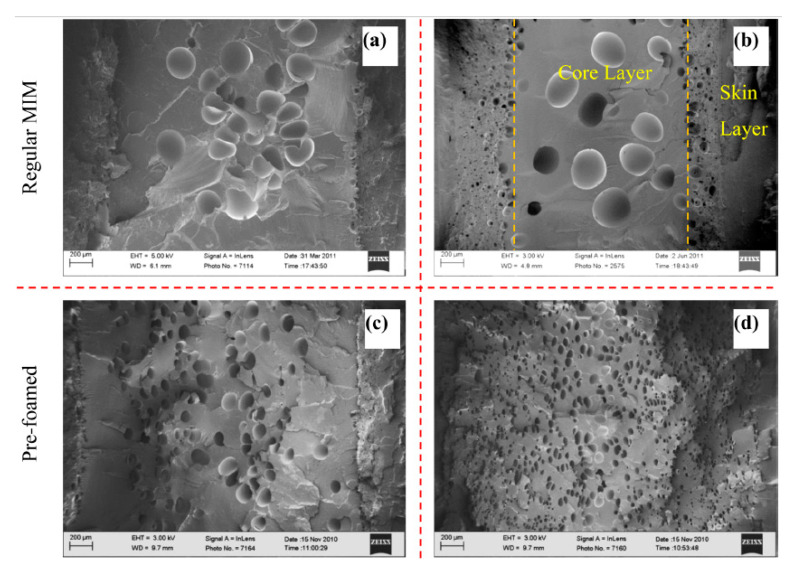
Cellular structures of (100× magnification): (**a**) neat PLA foam without pre-foaming, (**b**) PLA/clay foam without pre-foaming, (**c**) neat PLA foam with pre-foaming, and (**d**) PLA/clay foam with pre-foaming. Reprinted from Zhao et al. [90]. Copyright (2020) American Chemical Society.

Morphologies of foamed specimens with and without a pre-foaming step are shown in in Figure 27. Compared to neat PLA foams, the PLA/clays foams exhibited a better cell morphology. It was observed that the microstructure of the PLA/clay foam without pre-foaming was characterized by small cells near the skin layer, whereas larger pores could be found towards the center. The cell sizes in the skin layer were ~1 order of magnitude smaller than those in the core layer.

The cell sizes of the samples with pre-foaming were smaller and better distributed than those of the samples without pre-foaming. The cell morphology was improved when pre-foamed extrusion pellets were used for microcellular injection molding due to the co-blowing agent effect obtained with CO_2_ and N_2_ [64]. The nanocomposite foams had the smallest cell sizes and highest cell densities (5 μm and 1.5 × 10^9^ cells/cm^3^) and showed a microstructure in which the cells were round in shape, closed, and well-separated.

### 3.3. Conclusions

Two different types of FIM were presented in this section: high and low pressure. Nitrogen appeared as the common blowing agent. In the case of high-pressure FIM, opening the mold at slow speeds allowed to obtain cellular densities of the order of 2.2 × 10^8^ cell/cm^3^and small cell sizes of ~3 µm, with or without filler in the polymer matrix. This evidences the primary role of the operating conditions for controlling foam morphology.

The findings of Xie et al. [136], Zhao et al. [90], Najafi et al. [144], and Ding et al. [138] revealed that regardless of the filler and the blowing agent, foams with a “sandwich” structure are obtained when using the foaming injection molding process. A poorly foamed skin layer and a core with different cell morphologies could be identified in all foam samples. This confirms that the structure is inherent to the FIM process. High temperature and low shear exposure occurring in the core versus high speed cooling and high shearing occurring in the skin seem to be the main reasons explaining the difference between the skin and core morphologies. Note that the morphologies observed in the core are not always homogeneous. This has to be related with the interactions between the filler and the polymer matrix as well as the operating conditions used during the process.

The reviewed works allow assuming that the effect of the fillers is the same at both high and low pressure. When adding a particle, whatever its size and shape, higher cell densities and lower cell sizes were obtained. Nevertheless, not all the fillers will affect the morphology in the same quantitative way. One of the principal reasons for this is that depending on their characteristics they will not have the same effect on PLA’s crystallization kinetics. Ding et al. [138] highlighted the importance of the chemical and physical characteristics of the fillers.

## 4. Extrusion Foaming

**Figure 28:**
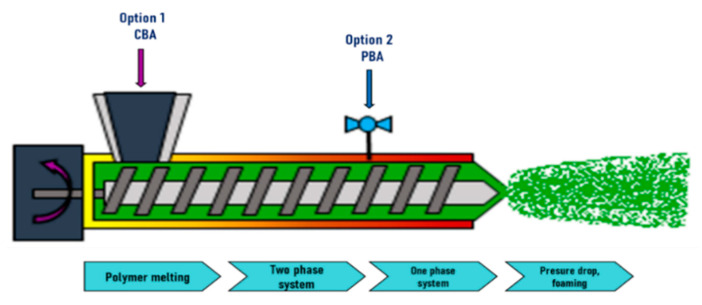
Principle of extrusion foaming. Adapted from Standau et al. [132].

It was in 1931 when Georg Munters and John Gudbrand [147] developed the first idea of polymer foaming using an extrusion process. In that first attempt, polystyrene was foamed using methyl chloride as the blowing agent. However, it appears that the major developments were led in the 1960s in the United States and Europe, and product platforms flourished in the 1970s. Generally speaking, thermoplastic foam extrusion matured in the 1990s [148]. A sketch of the foam extrusion process with one extruder is shown in Figure 28. The polymer in the form of pellets is fed into the hopper and is conveyed by the screw(s). As in the FIM process, blowing agents can be added through the hopper as shown in Figure 28 option 1 (in the case of chemical blowing agents) or at injection points in the barrel, option 2 (in the case of physical blowing agents). Inside the barrel the polymer melts and blowing agents are subjected to high pressure, and therefore the gas dissolves in the melt, leading to a single-phase homogeneous mixture. As can be expected, the gas-loaded melt can be further cooled through the extruder in all its residence time. This causes an increase in its viscosity, which in turn increases the pressure. Thereby, when going out through the die, a sudden pressure drop takes place. Here, the foaming phenomenon occurs, starting with cell nucleation and later cell growth. A stabilization step can be added, cooling with liquid (normally water) the produced foams just after the die. The extruded foams are limited in their geometry, which depends on the die shape (e.g., hole, slit, or ring die) [132].

The tandem line is a system developed to allow the prior dispersion of the PBA and enhance its mixing with the polymer [149]. This system is constituted by a first extruder having a single mixing screw and a second one having a cooling screw equipped with a gear pump, a heat exchanger, and a die. The first extruder plasticizes the polymer by dissolving the blowing agent. The gear pump provides the flow that is independent of temperature and pressure. The second extruder allows additional mixing and begins cooling, finally the heat exchanger removes the remaining heat.

### 4.1. Influence of Die Temperature

**Figure 29:**
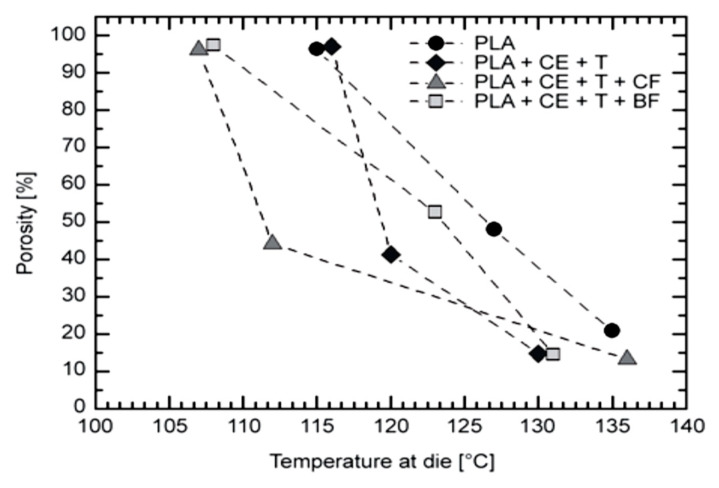
Effect of formulation and mixer and die temperatures on the porosity of PLA foams extruded at 8% of CO_2_. Reprinted from Bocz et al. [150] with the permission of Express Polymer Letters.

Die temperature has been proven to be one of the most important determinants of foam cell morphology [96]. Bocz et al. [150] foamed PLA, PLA/basalt (5 wt%), and PLA/cellulose (5 wt%) composites adding a (CE) chain extender (2 wt%) and talc as a nucleation agent (2 wt%). CO_2_ was used as the blowing agent. Figure 29 illustrates the effects of die temperature on foam porosity; it can be noted, in all cases, the lower the die temperature, the higher the porosity. Mihai et al. [92] reported this behavior to be linked with a skin formation at the surface of the samples due to lower die temperatures and an optimal melt temperature before the die. This frozen surface prevented CO_2_ from escaping, leading to pore growth and a higher expansion ratio. Compared to neat PLA, in the case of the additive containing mixtures, less porous foams were obtained at all die temperatures, indicating more gas loss when CE, talc, and fiber were present in the polymer melts. When decreasing the die temperature, a sharp increase in the porosity of the CE- and talc-containing PLA foam (PLA + CE + T) was observable; a similar behavior was observed for the cellulose containing mixture, indicating enhanced nucleation effect of the dispersed cellulose fibers.

**Figure 30:**
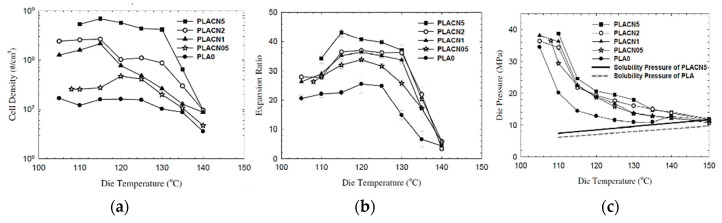
(**a**) Cell density and (**b**) expansion ratio of PLA & PLA/Cloisite 30 (PLACN) foams processed at various die temperatures, and (**c**) die pressure and solubility pressure at various die temperatures. Reprinted from Keshtkar et al. [151]. Copyright (2020), with permission from Elsevier.

As can be expected, not only can porosity be affected by die temperature, different characteristics of cell morphology can also be tuned by changing the die temperature. Keshtkar et al. [151] foamed PLA/Cloisite 30 (PLACN at 5, 2, 1, and 0.5 wt%) nanocomposites using CO_2_ as the blowing agent in a tandem line. In Figure 30a, it can be observed that cell density increased drastically (up to two orders of magnitude) when using lower die temperatures. Figure 30c shows that at all die temperatures, the die pressure was higher than the CO_2_ solubility pressure for PLA and PLA composites. This indicates that the PLA/gas phase separation had been avoided before foaming. In Figure 30b, it can be observed that the effect of the die temperature presents a threshold value, for which further decrease of die temperature causes a decrease of the expansion ratio. This is because at too low a temperature the polymer becomes stiffer and the time available for the growing of the cells is limited, provoking a low expansion ratio. There is thus an optimal die temperature to reach the highest cell density and expansion ratio.

### 4.2. Temperature Profile Effects

Keshtkar et al. [151] showed that when die temperature is high, temperature profile does not have an important role on cell morphology. On the other hand, at lower die temperature, controlling the temperature profile can help to tune the cell morphology. Keshtkar et al. [151] tested three different temperature profiles in the second extruder of a tandem line as indicated in Table 4, and the die temperature was maintained at 120 °C. Neat PLA and two PLA/clays (PLACN 1, 5 wt%) nanocomposites were foamed.

**Figure 31:**
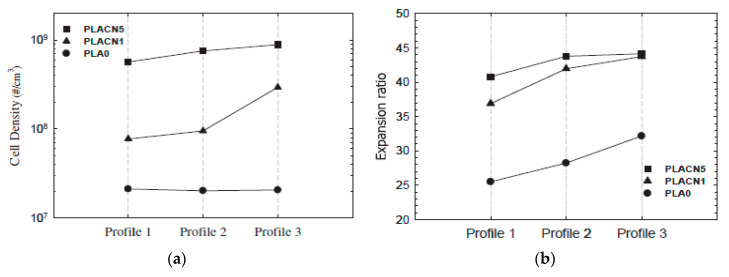
(**a**) Cell density and (**b**) expansion ratio of foams obtained by varying temperature profiles in the second extruder of tandem line foaming extrusion system from neat PLA (PLA0) and PLA with 1 wt% (PLACN1) and 5 wt% (PLACN5) of Cloisite 30. Reprinted from Keshtkar et al. [151]. Copyright (2020), with permission from Elsevier.

Figure 31 Profile 3 promoted the cell density and the expansion ratios of the PLA and PLACN sample, notice that profile 3 corresponds to the one with lower temperatures and could have accelerated the isothermal crystallization of PLA and PLACN along the extruder. Therefore, by choosing a lower temperature profile, it could be expected enhanced crystallization kinetics and thereby higher cell nucleation rate through promoted heterogeneous cell nucleation around the crystals and increased expansion ratio via improved melt strength.

### 4.3. CO_2_ Concentration Effects

**Figure 32:**
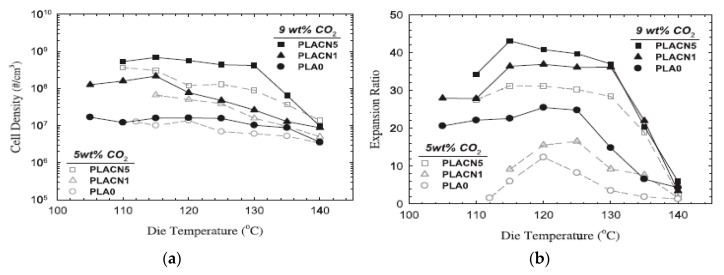
Comparison of (**a**) cell density and (**b**) expansion ratio of foams obtained at various die temperatures with 5 and 9 wt% CO_2_ from neat PLA (PLA0) and PLA with 1 wt% (PLACN1) and 5 wt% (PLACN5) of Cloisite 30. Reprinted from Keshtkar et al. [151]. Copyright (2020), with permission from Elsevier.

Keshtkar et al. [151] also demonstrated that regardless of the die temperature, PLA and PLA/Cloisite 30 (PLACN) foams showed higher expansion and higher cell density at higher CO_2_ concentration (Figure 32). A more uniform foam morphology was obtained when up to 9 wt% of CO_2_ injected in the barrel was used. At high CO_2_ content, a greater degree of thermodynamic instability is induced, and this results in a larger cell density. Moreover, the increased gas content might also have enhanced the crystal nucleation rate along the second extruder, due to the cooling that occurs throughout the extruder and to the reduced dissipation energy required for crystallization through CO_2’_s plasticization effect [114]. It is worth mentioning that as suggested by Bocz et al. [150], the effect of CO_2_ content is also intimately related to the temperature of the polymer melt as the CO_2_ solubilization is inversely proportional to temperature. Thereby, high dissolved CO_2_ content can only be obtained at low temperatures.

### 4.4. Fillers Content and Shape Effects

As discussed for batch and FIM processes, the presence of different fillers influences the foamability of the composites by modifying their rheological properties and its crystallisation behaviour. With the addition and dispersion of fillers such as clays, the PLA melt strength is increased, and thereby the foaming behaviour can be significantly improved. Liu et al. [152] studied the effect of the clay content on the cell morphology of PLA/Clay foams. The used organoclay, under the commercial name of I.34TCN from Nanomer Products Inc., was organics-treated by a quaternary ammonium ion containing methyl tallow bis-2-hydroxyethyl. In this case, a chain extender (CESA extend BL 10,069 N) was used at a content of 5 phr (per hundred resin) in all the samples as well as an antioxygen (Irganox1010) at 0.1 phr. Three different clay contents were studied 1, 3 and 5 phr. In general, the foamed composites showed smaller cell size and larger cell density compared to neat PLA samples, but nanocomposites with too high organoclay content (>3 phr) were poorly foamed due to the poor dispersion and exfoliation of the clays. In the study of Keshtkar et al. [151], increasing Cloisite 30B contents led to both enhanced expansion ratio and cell density of the foam samples. Even at a clay content of 5 wt%, higher expansion ratios with finer cells were obtained together with a wider processing window. In these two studies, the effect of “high” clay content was thus not the same [150,151,152], highlighting that not only the operating conditions are relevant, but also the chemical and physical properties of the fillers. It has to be said that the nature of the PLA and the interactions with additives are important too and cannot be neglected. Keshtkar et al. [151], also demonstrated that for Cloisite 30B, the quality of the dispersion played a key role in the final foam cell density and cell size.

**Figure 33:**
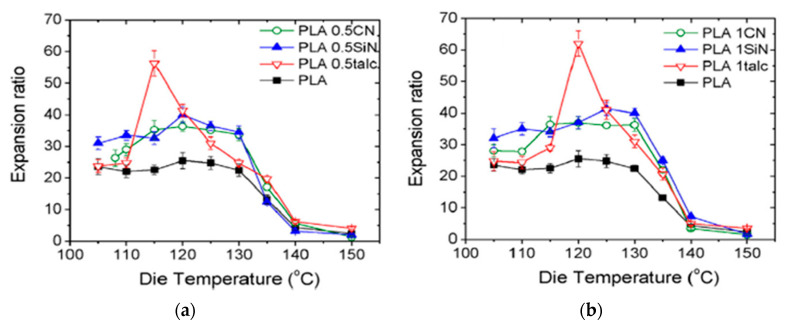
Expansion ratio of the foamed PLA and PLA/Clay, PLA/Silica and PLA/Talc composites at various die temperatures (**a**) Content of 0.5 wt% and (**b**) Content of 1 wt%. Reprinted from Nofar [113]. Copyright (2020), with permission from Elsevier.

Nofar [113] carried out experiments at different die temperatures in the second extruder of a tandem line. This work allowed to compare the effect of different fillers at various contents. Cloisite 30B, Nano silica Aerosil A200, and Mistron Vapor- R grade talc were used. Nanoclay and Nano silica were referred to as CN and SiN, respectively. In PLA nano/micro composites, the addition of only 0.5 wt% of each additive significantly increased the cell density of the PLA foams. However, although in PLA-talc samples, the number of talc particles was smaller than the number of nanoparticles in PLA nanocomposites at the same content, the obtained cell density in PLA-talc foamed samples was very similar to that obtained in the PLA-nanocomposite foams. Authors suggest that talc had a better performance as a nucleation agent than the other fillers, but it should be noted that due to the strong effect of nanoparticles in the viscosity of the composites, the expansion of the foams can be limited and the cell density can be impacted. Figure 33 shows that a high die temperature, the fillers’ effect on expansion ratio is negligible, which can be explained because at high temperatures, the gas loss is incremented, therefore the gas available for the expansion is lower. At low temperatures, the effect of the fillers is almost the same, this is maybe due to the polymer hardening at these temperatures. The expansion ratio behaviour of the foamed PLA nano/micro composites with 1 wt% of the additives showed a similar trend to that of the foamed PLA samples with 0.5 wt% of the additives. Suggesting that the fillers’ content has not an influence in the way that expansion ratio changes with temperature.

**Figure 34:**
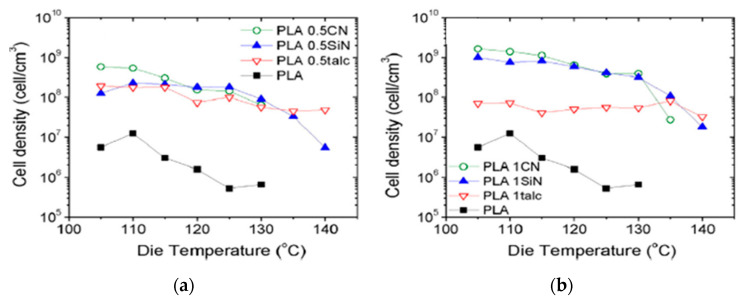
The cell density of foamed PLA and PLA/clay, PLA/silica, and PLA/talc composites at various die temperatures, (**a**) content of 0.5 wt% and (**b**) content of 1 wt%. Reprinted from Nofar [113]. Copyright (2020), with permission from Elsevier.

When the additive content was increased from 0.5 wt% to 1 wt%, a further enhancement in the cell density of the PLA foams was expected. As Figure 34 shows, the increased nanoparticle content significantly promoted the cell density of the PLA foamed samples. On the other hand, when the talc content was increased, the cell density was somehow decreased and the cell morphology showed less uniformity than neat PLA foams. This was despite the larger number of available cell nucleating sites that were supposed to enhance the cell density and morphology. A possible reason can be illustrated by the enhanced crystallization kinetics of PLA during the foaming process when increasing the talc content at various die temperatures as explained by Nofar et al. [114]. This resulted in a different CO_2_-solubility profile within the polymer–CO_2_ mixture. Consequently, the polymer–CO_2_ phase separation caused by the faster crystallization further decreased the die pressure and increased the rigidity of PLA. This decrease in the die pressure implied a smaller pressure drop, affecting the thermodynamic instability in the system and hence the cell density.

**Figure 35:**
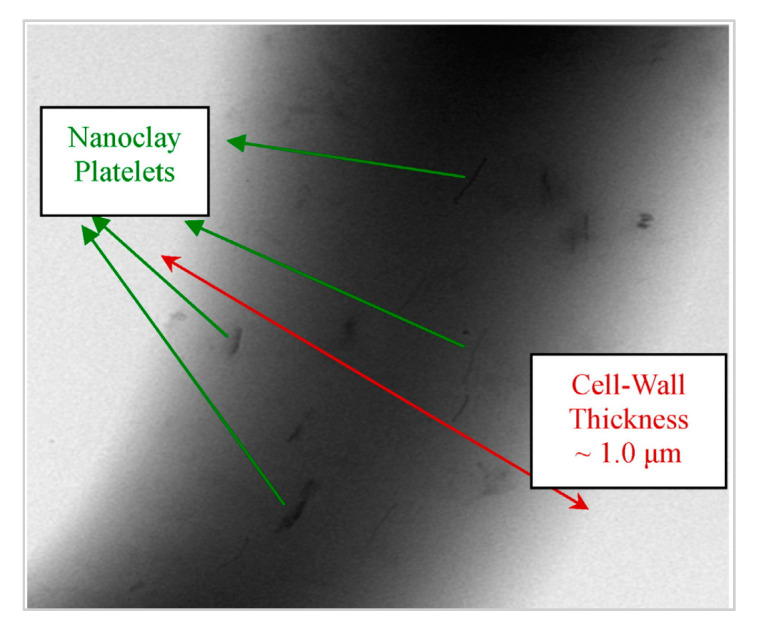
TEM image of a cell wall in foamed PLA-1CN showing the alignment of nanoclay platelets along a cell wall. Reprinted from Nofar [113]. Copyright (2020), with permission from Elsevier.

Nofar [113] also compared the effect of nanoclay and nanosilica particle shape. The fillers had differences in their geometries and aspect ratios: nanosilica particles had a mean particle size of 12 nm and the nanoclay platelets had an average width of 100 nm and thickness of 1 nm for each lamella. Despite their similar influence on the final expansion ratio and cell density of the foamed samples, a more closed cell structure was obtained in the PLA-CN foam samples compared with the PLA-SiN samples. The authors suggest that the platelet-shaped nanoclay with a long two-dimensional aspect ratio must have been oriented along the cell walls due to the biaxial stretching occurring during the foam expansion as demonstrated by Okamoto et al. [153] in polypropylene/clay nanocomposite foams. Therefore, the cell rupture could be inhibited in a greater measure in PLA-CN foamed samples than in PLA with three-dimensional bulk nanosilica particles due to the increased cell wall strength. Figure 35 shows a TEM image of a single cell wall in the PLA-1CN foamed sample which reveals the alignment of the long aspect ratio nanoclay platelets along the cell wall.

### 4.5. Chain Extender Effects

**Figure 36:**
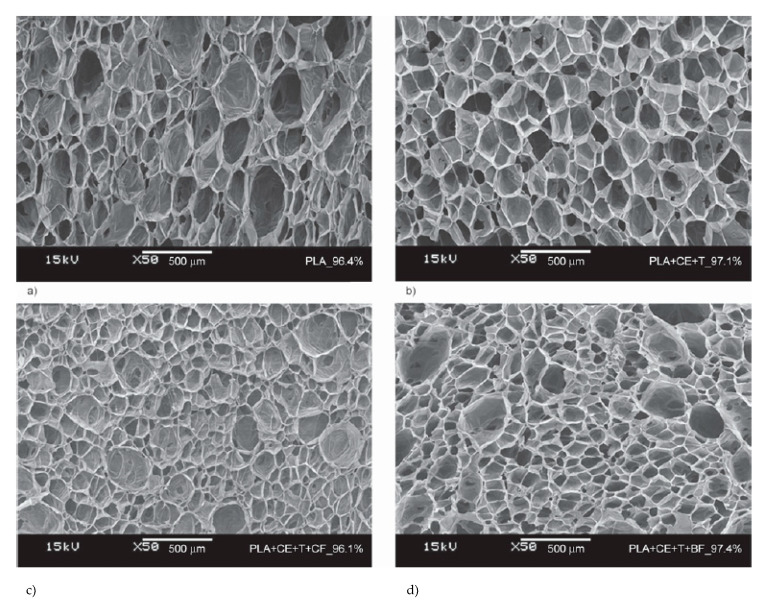
SEM micrographs of the cell morphologies obtained for highly expanded (ε > 95%) PLA foams. (**a**) PLA, (**b**) PLA + CE + T, (**c**) PLA + CE + T + CF, and (**d**) PLA + CE + T + BF. Reprinted from Bocz et al. [150] with permission from Express Polymer Letters.

It is well known that the low melt strength of PLA is one of the obstacles to obtaining well-foamed PLA samples. Introducing a chain extender to create a branched structure [97,98,99] is one of the strategies to improve the melt strength of PLA as well as the use of different fillers in order to control its rheological properties. Bocz et al. [150] studied the effects of adding a CE to improve the foamability of PLA, PLA/cellulose, and PLA/basalt samples at a content of 5 wt%. Talc was used as a nucleation agent. Joncryl ADR4368-C was used at a content of 2 wt% in all samples. With neat PLA, a broad cell size distribution with limp or collapsed cell walls was observed. The evidence that the addition of CE effectively increased the melt strength is based on the fact that the PLA foam containing it and talc (PLA + CE + T) had a denser and more uniform cell morphology. The samples obtained by Bocz et al. [150] are shown in Figure 36.

From the findings of Bocz et al. [150], it can be assumed that the filler can affect the efficiency of the CE to improve foaming. On the other hand, Rokkonen et al. [154] also found that the CE can in turn affect the effects of the blowing agent during the foaming process. Bleached hardwood (birch) Kraft pulp fibers were used as filler at contents of 10 and 20 wt%. Joncryl 4368-CS was used as a chain extender at 0.7 wt%, and talc was used as nucleation agent at 0.1 wt%. In the foaming process, isobutane and sc-CO_2_ were used as blowing agents. Samples loaded with fibers and talc as well as samples loaded with fibers, talc, and CE were foamed. First of all, a lack of CE led to either collapsed cell structures or severe damages within the cell structure. It was also found that the addition of CE could affect the foaming temperature. Indeed, when using CO_2_ as the blowing agent, lower foaming temperatures could be used compared to the isobutane due to its plasticization effect. In all cases, when adding the chain extender, the minimum foaming temperatures increased by 10 °C for all the samples and both foaming agents.

A different approach was developed by Matuana and Díaz [155] to improve the foamability of PLA/Wood composites. This work was based on the hypothesis that MFI of the molten polymer or composite could be a key parameter that determines the quality of the foam structure. On this basis, a composite adjusted to the same MFI as a PLA having a good foamability, could be foamed with a comparable cell morphology. Epolene-E43 was used as rheology modifier; talc at 2 wt% was used as a nucleation agent. Composites with wood flour contents of 10–20 and 30 wt% were produced. The optimum concentration of E-43 for each wood flour content was adjusted by measuring the MFI of each sample at different E-43 contents. The target content was defined when the sample matched the MFI of pure PLA (6.9 g/10 min). In all cases, it was observed that the MFI increased with the E-43 content and that the E-43 content must be adjusted according to the wood flour content, i.e., the higher the wood flour content, the lower the E-43 content.

**Figure 37:**
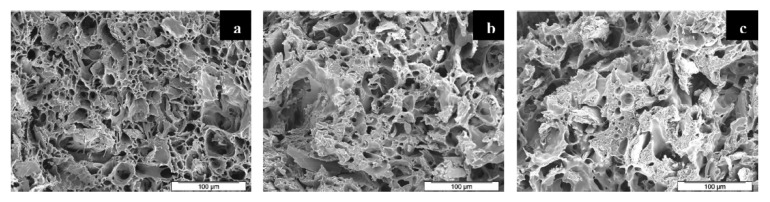
Effect of rheology modifier content on cell morphology of foamed PLA samples containing 20 wt% wood flour: (**a**) 2 wt%, (**b**) 4 wt%, and (**c**) 6 wt% E-43. Reprinted from Matuana and Díaz [155]. Copyright (2020) American Chemical Society.

Figure 37 shows the foamed samples having 20 wt% of wood flour and increasing E-43 contents, corresponding to decreasing MFIs. Below the matching point (1.3 wt% E-43), and due to the high melt viscosity (i.e., lower MFI value of 6.1 g/10 min), the extrusion foaming process was unstable. In contrast, with values closer to and above to that of unfilled PLA (i.e., 2, 4, and 6 wt% E-43), the foaming process of composites was successful. When the composite′s MFI was closer to the matching point, foams with uniform and homogeneous cell structures were obtained (see Figure 37a). For the composites containing 2 wt% E-43, it could be possible that the melt viscosity was low enough to allow cell formation and growth while being high enough to prevent cell coalescence. Cell structures of foamed composites slightly deteriorated above the matching point (4 and 6 wt% E-43), showing large cracks and cell coalescence (Figure 37b,c)). Indeed, the melt viscosity was excessively lowered (higher MFI) and provided insufficient melt strength to trap the growing bubbles and avoid cells coalescence. These results depicted the importance of the melt flow index of the polymers in an extrusion foaming process and show that the cell morphology can be varied and improved by tuning the MFI.

### 4.6. Conclusions

The quality of the foams produced by the extrusion foaming process is greatly influenced by the operating temperatures. The most important parameter is the die temperature, i.e., low die temperatures favor a uniform cell morphology and generate foams with high cell density and small cell sizes. The different results found in the literature suggest that an optimal die temperature can be determined for each PLA/additives system, allowing to obtain the best combination of foam characteristics. Die temperatures ranging between 150 °C and 115 °C were used and cell densities from 1 × 10^6^ cell/cm^3^ until 1 × 10^9^ cell/cm^3^ were obtained. Along with the die temperature, lowering the temperature profile in the extruder should also be favored, lower temperatures enhancing the PLA crystallization and promoting cell nucleation during the foaming step. In addition, the solubility of the physical blowing agent, usually carbon dioxide, is increased by lowering temperature of the polymer melt. Besides, processing parameters as screw speed and screw profile can affect the shearing and mixing of the components during the extrusion foaming and influence to a lesser extent the properties of the produced foams.

When working with a polymer with a low melt strength such as PLA, chain extenders and fillers are employed as strategies to improve this rheological property and control the foam morphology. Higher cell densities and smaller cell sizes can be reached when fillers are added in the PLA matrix. However, the foam quality and filler content can vary depending on the chemical and physical characteristics of the filler, i.e., size, shape, and surface chemistry. In the reviewed works, filler contents from 0.5 wt% to 30 wt% for microparticles and from 0.5 wt% to 5 wt% for nanoparticles were used. The matrix–filler and filler–filler interactions, and therefore filler dispersion, are also key parameters for producing a foam with the desired cell morphology. When a chain extender is added to pure PLA, an improvement in the cell morphology is obtained due to enhanced melt strength. On the other hand, when using both chain extender and fillers, the latter can interact together as well as with PLA itself and hamper the foaming. The amount and type of chain extender should thus be tuned according to the filler type and content.

Finally, it must be pointed out that extrusion foaming is a good option for producing PLA foams in a continuous process with desired morphology and properties, whose can be tuned by changing the formulation and operating conditions.

## 5. Conclusions

Developing PLA/nano- and microcomposites appears as an efficient and “green” pathway to improve the PLA foamability. Foams with better cell morphology than those obtained with neat PLA ones can be achieved, regardless of the implemented process. This technique is easy to apply and is versatile due to the variability of fillers that can be used (e.g., lignocellulosic and mineral) and the opportunity of combining it with other additives as chain extenders or with filler surface treatments. While the foaming of PLA has been studied in-depth and the operating conditions of each supercritical fluid assisted process have been well evaluated, the role of the filler on the foaming process and foam morphology needs to be deeper investigated and understood. Generally speaking, fillers change the cell structure and mechanical properties of the foams, but each type of filler has specific effects depending on its characteristics. This review highlighted that the physical properties of fillers, such as their size and shape factor, but also their surface chemistry, can have an important influence on the final cell morphology, but currently these properties have not yet been a field of research explored in much detail. A better understanding of the filler’s impact on microcellular polymers can lead to the production of foams with well-controlled morphologies and functional properties for different application fields.

## Figures and Tables

**Table 1:** Operating conditions used in the batch foaming process for polylactic acid (PLA)-based (nano) biocomposites.

Filler	Content wt%	Pressure MPa	Temperature ^1^ °C	Temperature ^2^ °C	Time ^3^ h	Ref
**Temperature Induced Foaming**						
Nano cellulose (CNF)	0.5-1-3	4.14	25	100	24	[120]
Micro sized cellulose	0.5-1-3-5-10	4.14	25	100	24	
Cellulose nanocrystal (CNC)	3	5	25	60	12	[121]
Chitin	1-2-5	4.14	25	95	24	[122]
Nano chitin	1-2-5	4.14	25	95	24	
Wood flour	10-20-30-40	2.76	25	150	96	[123]
**Pressure Induced Foaming**						
MFC bleached softwood pulp	1-5	20	190		0.3	[124]
Nano cellulose	1-2-5	20			1	[125]
Bleached Kraft pulp (Nano cellulose)	2,7-9	12-13-14-16-18-20	60		6	[126]
Bleached birch Kraft pulp (Wood fibers)	1-5-10	20	185		0.6	[127]
Jute microfibers	5-10-20-30	17	150		6	[128]
Silk	1-3-5-7	20	135-145-165-175		1	[129]
Wood flour	20	16-11	180-100		0.6	[130]
Organically modified layered silicate	4	14-18-21-24-28-30	100-110-120-130-140-150			[131]

^1^ Saturation temperature; in the pressure-induced foaming, saturation and foaming temperature are the same. ^2^ Foaming temperature.^3^ Saturation time.

**Table 2:** PLA composites by foam injection molding (FIM) technique and their operating conditions.

Filler	Supercritical Blowing Agent				Mold Temperature°C	Melt Temperature°C	Injection Speed	Ref
	Nature	PressureMPa	Contentwt%	Flow Ratekg/h				
Clays	N_2_/CO_2_	30		0.11	10	180	20 cm^3^/s	[90]
Flax	N_2_			0.08	20	185	20 cm^3^/s	[105]
Clays	N_2_	12	0.6		25	185	100 mm/s	[136]
Willow	N_2_	19.3	0.69	0.19	24	200	35–45 mm/s	[137]
Cellulose	N_2_		0.5		40	170	100 cm^3^/s	[138]

**Table 3:** Characteristics of Northern bleached softwood Kraft (NBSK) and medium density fiberboard (MDF) cellulosic fibers. Reprinted from Ding et al. [138]. Copyright (2020), with permission from Elsevier.

Fiber Type	Length (mm)	Shape Factor	Cellulose (%)	Hemicelluloses (%)	Lignin (%)	Extractives (%)
NBSK	2.20	>60	97.50	0.50	<2	0.03
MDF	2.19	26	47.03	6.86	25.7	16.0

**Table 4:** Various temperature profiles during extrusion foaming in the second cooling extruder of the tandem-screw extruder. Reprinted from Keshtkar et al. [151]. Copyright (2020), with permission from Elsevier.

	Zone 1	Zone 2	Zone 3	Heat Exchanger	Die
Profile 1	180	140	130	130	120
Profile 2	180	135	125	125	120
Profile 3	180	130	120	120	120
